# Expression and prognosis analyses of the fibronectin type-III domain-containing (FNDC) protein family in human cancers: A Review

**DOI:** 10.1097/MD.0000000000031854

**Published:** 2022-12-09

**Authors:** Hui Jiang, Bo Ling Chu, Jiao He, Zhi Liu, Ling Yang

**Affiliations:** a Biobank of Pathology Department, Suining Central Hospital, Suining, Sichuan, China; b Department of Pathology, Suining Central Hospital, Suining, Sichuan, China.

**Keywords:** cancer, FNDC, mRNA expression, prognosis

## Abstract

Despite advancements in early detection and treatment, cancer continues to pose a threat to human health and is the leading cause of death worldwide. According to recent research, the fibronectin type-III domain-containing (FNDC) protein family has been implicated in several different human disorders. However, little is known regarding their expression and prognostic significance in most human malignancies. We carried out a thorough cancer vs. normal expression study using the Oncomine and Tumor Immune Estimation Resource (TIMER) databases, as well as a prognostic evaluation using the Kaplan-Meier (KM) plotter and PrognoScan databases. Oncomine revealed that the mRNA expression levels of FNDC1, FNDC3A, and FNDC3B were higher in most malignancies than in normal tissues, but the mRNA expression levels of FNDC4, FNDC5, FNDC7, and FNDC8 were downregulated in most cancers when compared with normal tissues. In survival analyses based on KM Plotter and PrognoScan, all members of the FNDC family displayed significant correlations with survival outcomes in breast, gastric, and ovarian cancers. Furthermore, the whole FNDC family, except for FNDC7 and FNDC8, was found to have substantial predictive effects in lung adenocarcinoma, but not in squamous cell lung cancer. In addition, potential connections between several FNDC family members and survival results in liver and colorectal malignancies were discovered in this study. One or more members of the FNDC family demonstrated statistically significant differences in expression between cancer and normal tissues, suggesting that they could be used as prognostic biomarkers for specific cancers.

## 1. Introduction

Cancer is the second most common cause of death and is a serious public health concern worldwide. It is predicted that there will increase of 27.5 million new cancer diagnoses each year by 2040 worldwide, from 17 million in 2018.^[1]^ Due to the aging population, the trend toward environmental deterioration, and the appearance of some undesirable behaviors and habits, the burden of cancer has increased considerably worldwide, even with advancements in cancer diagnosis and treatment.^[2]^ Therefore, there is a pressing need to investigate the fundamental causes of cancer and develop possible biomarkers to enhance the diagnosis, treatment, and prognosis of patients with cancer.

Fibronectin type III domain-containing (FNDC) proteins are distinguished by the presence of at least 1 conserved fibronectin type III domain of fibronectin, an important type of extracellular matrix protein and a well-known tumorigenesis regulator.^[3]^ From the UniProt Knowledgebase, the FNDC protein family contains 11 proteins in humans, namely FNDC1, FNDC3A, FNDC3B, FNDC4, FNDC5, FNDC6 interleukin-20 receptor subunit beta (IL20RB), FNDC7, FNDC8, FNDC9, FNDC10, and FNDC11. Tissue development, cell adhesion, migration, and proliferation are among the many roles they perform.^[4,5]^ Some studies have also shown that FNDCs are regulated by microRNAs^[6–8]^ and circRNA.^[9]^ Other processes, such as the regulation of adipogenesis and osteoblast differentiation by FNDC3B,^[10]^ regulation of FNDC4 by TGF-*β*,^[11]^ and involvement of the corticoid receptor in the regulation of FNDC5^[12]^ expression, have also been discovered. General expression analyses and functional findings are available for the genes FNDC1, FNDC3A, FNDC3B, FNDC4, FNDC5, and FNDC6. FNDC1 was discovered to be a regulator of cardiovascular function and to play a role in the death of cardiomyocytes triggered by hypoxia in 1 study.^[13,14]^ Moreover, FNDC1 has been linked to an increased risk of arterial hypertension according to a quantitative trait loci study.^[15]^ In contrast, the expression of FNDC1 was correlated with tumorigenesis in gastric,^[16]^ breast,^[17]^ and prostate^[18]^ cancers. Expression analyses showed that FNDC3A was upregulated in CRC,^[4]^ and downregulation of FNDC3A expression reduced the ability of cervical cancer cells to proliferate, migrate, and colonize their environment.^[19]^ Epithelial-to-mesenchymal transition is accelerated by FNDC3B, which activates various pathways in various cancer cells, including hepatocellular carcinoma,^[5]^ acute promyelocytic leukemia,^[20]^ and tongue squamous cell carcinoma cells.^[21]^ FNDC4 is an anti-inflammatory factor that is upregulated in patients with inflammatory bowel disease.^[4]^ Furthermore, FNDC4 operates as an extracellular factor that increases the invasiveness of HCC, in part by activating the PI3K/Akt signaling pathway, as previously described.^[22]^ To date, FNDC5 has been the most extensively researched FNDC primarily because it transports the peptide hormone irisin, which has been postulated to stimulate adipose tissue conversion.^[23]^ Interleukin-20 receptor subunit beta (IL20RB/FNDC6) is a type II cytokine receptor with a single-pass type I membrane protein, which plays a role in the pathophysiology of chronic inflammation and autoimmune disorders. However, little is known about FNDC6’s role in cancer. For example, in papillary renal cell cancer, FNDC6 overexpression is linked to increased migration and poor prognosis.^[24]^

In brief, these findings suggest that some cancers can choose members of the FNDC family as prognostic biomarkers or potential therapeutic targets. However, a comprehensive analysis of the transcriptional expression and prognostic relevance of FNDC family members in human malignancies is currently lacking. In this investigation, we 1^st^ examined the changes in mRNA expression of FNDC family members between cancer and normal tissues in common human tumors using the Oncomine and tumor immune estimation resource (TIMER) databases to determine if there were any differences. Second, we examined the prognostic value using the Kaplan-Meier (KM) plotter and PrognoScan databases.

## 2. Material and Methods

### 2.1. Oncomine database analysis

Oncomine (https://www.oncomine.org/resource/login.html),^[25]^ an online microarray database that offers the most complete spectrum of mutations, gene expression data, and clinical information, can be used to identify novel biomarkers or new treatment targets. Discrepancies in FNDC family mRNA expression in common human malignancies were examined using the Oncomine database. The thresholds for each malignancy and gene were as follows: the fold change was 2, the *P* value was 0.01, the gene rank was in the top 10%, and the data type was mRNA. The cancers, datasets, genes, sample sizes, *t* test, fold change, and *P* value were derived from studies that indicated statistical differences. Thus, the Oncomine data platform was terminated on January 17, 2022. Therefore, we also studied the transcriptional expression of FNDC family members in the TIMER database.

### 2.2. TIMER database analysis

It is possible to determine the clinical relevance between distinct immune cells and different cancer types using TIMER (http://timer.cistrome.org/),^[26]^ which is an excellent online tool. Using a novel statistical method, TIMER calculates the number of immune cells in the tumor microenvironment: B cells, CD4 T lymphocytes, CD8 T lymphocytes, neutrophils, macrophages, and dendritic cells. These findings were confirmed through pathological examinations. Gene expression and survival analyses were performed using TIMER. The current version of TIMER is available at TIMER2.0, and it contains 10009 samples from TCGA representing 23 different cancer types.

### 2.3. Kaplan–Meier plotter database analysis

To assess the prognostic roles of the FNDC family in these cancers, we used the KM plotter (http://kmplot.com/analysis/),^[27]^ an online prognostic assessment database that can assess the effect of 54k genes (proteins, DNA, mRNA, and miRNA) on survival in 21 forms of cancer, including 7830 breast cancers, 2190 ovarian cancers, 1440 gastric cancers, and 3452 lung cancers. Based on the intermediate levels of mRNA expression, cancer patients were separated into 2 groups: those with high expression and those with low expression. For each gene, the required probe ID was placed in the database separately for each high-and low-expression group. Statistical significance was be reached *P* value < .01. It was possible to summarize the information from the KM plotter homepage in terms of cancer type, Affymetrix ID, genes, survival results (with 95 percent confidence intervals), HRs, and *P* value; some typical plots were also provided.

### 2.4. PrognoScan database analysis

The following qualities describe the PrognoScan database: a large-scale collection of data, a systematic analytic method for gene prognostic value, and tumor markers and proto-oncogenes that can be evaluated using this strong analytical framework. The PrognoScan database (http://www.abren.net/PrognoScan/)^[28]^ looks through a large number of publicly available cancer microarray datasets for correlations between gene expression and patient prognoses, such as overall survival (OS), relapse free survival (RFS), and disease free survival (DFS). The criterion was raised to a *P* value of < .05 for Cox regression. We chose datasets with a Cox *P* value of < .01 for research.

## 3. Results

### 3.1. The mRNA expression levels of the FNDC family in human tumors

**Figure 1.**
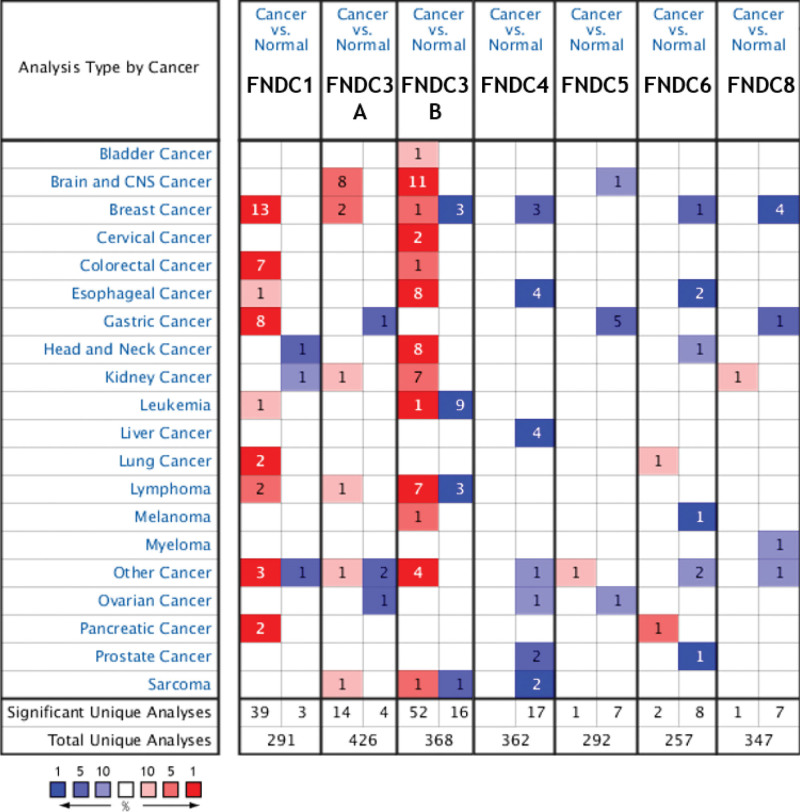
The mRNA expression levels of the FNDC family in human cancers. The number of analyses that meet the thresholds was shown in the colored cells. The gene rank determines the cell color, which represents the importance of genes in cancer. The red and blue indicate over-expressed and under-expressed respectively, and the brighter red or blue implies a gene with a higher or lower level of expression that is more statistically significant. FNDC = fibronectin type-III domain-containing, KM = Kaplan-Meier.

Oncomine was used to study changes in mRNA expression of the FNDC family in various types of cancerous and non-cancerous tissues. As illustrated in Figure 1, the database had a sum of 291, 426, 368, 362, 292, 257, and 347 unique analyses for FNDC1, FNDC3A, FNDC3B, FNDC4, FNDC5, FNDC6 (IL20RB), and FNDC8, respectively. Under the set threshold conditions, FNDC7 did not yield any results. To date, the Oncomine database does not contain any data for FNDC9, FNDC10, and FNDC11. Therefore, we analyzed the expression and prognosis of FNDC1, FNDC3A, FNDC3B, FNDC4, FNDC5, FNDC6, FNDC7, and FNDC8. In 42 studies, FNDC1 was placed in the top 10% of all genes, indicating a statistically significant difference. Of the 42 studies, 39 revealed higher expression levels in tumors than in normal tissues, with only 3 studies showing reverse outcomes. FNDC3A was shown to be upregulated in malignancies in 14 studies, while it was also found to be under-expressed in 4 studies. High expression of FNDC3B was discovered in 52 studies of 13 cancers, and its low expression was only found in 4 cancers and 16 studies. Seven tumors had low levels of FNDC4 mRNA expression, as demonstrated by 17 statistically significant and unique analyses. FNDC5 levels in tumors were lower than those in neighboring normal tissues, as demonstrated by 8 analyses involving 3 different types of cancer; only 1 analysis revealed an increased level. The expression of FNDC6 and FNDC8 was lower in cancerous tissues than in normal tissues in the majority of cases. Overall, FNDC1, FNDC3A, and FNDC3B had significantly higher levels of mRNA expression in most malignancies than in nearby normal tissues, whereas FNDC4, FNDC5, FNDC6, and FNDC8 had significantly lower levels of expression.

As the second largest cause of death in the world, the number of deaths and incidence of cancer is increasing every year. The number of global cancer cases may increase by 60% in the next 20 years, according to the World Health Organization’s assessment; thus, the prevention and control of cancer should not be slackened.^[7]^ The most common cancers worldwide are lung, breast, colorectal, prostate, gastric, and cervical. According to the 2015 China Cancer Report, lung cancer, gastric cancer, colorectal cancer, liver cancer, breast cancer, and esophageal cancer are the most prevalent malignancies in China, as opposed to the global cancer spectrum,^[29]^ which may be related to culture, dietary patterns, and low intake of nutrients in food and food contamination.^[30]^

For this reason, we were interested in the expression and prognosis of the FNDC family in these 4 tumor types, including breast, lung, gastric, and colorectal cancers, along with many other prevalent solid tumors such as ovarian cancer and liver cancer.

### 3.2. The expression level and prognostic value of the FNDC family in breast cancer

Oncomine was used to investigate the expression of the FNDC family of genes in ductal, tubular, and invasive breast carcinomas, and the results were promising. The analysis involved a total number of 27 data sets. For FNDC1, 13 of the 45 analyses showed significant differences between the groups of patients with cancer and healthy individuals. Following analysis of the Ma 4 dataset, we discovered that the FNDC1 gene was significantly expressed in 3 different types of breast cancer: ductal breast carcinoma in situ, invasive ductal breast carcinoma (IDC), and IDC epithelia. In the other 6 databases, we reached the same conclusion. According to the Radvanyi and Finak databases, FNDC3A expression is elevated in both invasive mixed and invasive breast carcinomas. Regarding FNDC3B, only 1 out of 34 analyses showed significantly increased expression between the cancer and non-cancerous groups. FNDC3B was shown to be overexpressed in invasive breast cancer, which was identified by the Finak dataset, whereas we obtained the opposite conclusion from other databases, including TCGA, Radvanyi, and Gluck. It was found that the level of FNDC4 expression in IDC and mixed lobular and ductal breast carcinoma was low, and this was also found in databases such as TCGA. According to TCGA database, FNDC6 was downregulated in intraductal cribriform breast adenocarcinoma. FNDC8 was decreased in ductal breast cancer in situ, invasive ductal and lobular breast cancer, and invasive mixed breast carcinoma in studies from TCGA database. For FNDC5, cancer tissues were not significantly different from the normal tissues. Table 1 contains a list of all the reports that were statistically significant.

**Table 1:** Datasets of FNDC family in breast cancer.

Gene	Dataset	Normal (Cases)	Tumor (Cases)	Fold change	*t* Test	*P* value
FNDC1	Ma 4	Breast (14)	Ductal Breast Carcinoma in Situ (11)	25.295	14.788	2.32E–13
		Breast (14)	Invasive Ductal Breast Carcinoma Stroma (9)	34.711	9.828	3.01E–7
		Breast (14)	Invasive Ductal Breast Carcinoma Epithelia (9)	2.552	3.796	.002
	TCGA	Breast (61)	Mucinous Breast Carcinoma (4)	**2.609**	7.688	2.14E–10
		Breast (61)	Mixed Lobular and Ductal Breast Carcinoma (7)	**2.285**	5.287	8.75E–6
		Breast (61)	Invasive Lobular Breast Carcinoma (36)	**2.804**	7.353	4.62E–11
		Breast (61)	Male Breast Carcinoma (3)	**2.616**	5.393	9.46E–4
		Breast (61)	Invasive Breast Carcinoma (76)	**2.897**	7.942	4.12E–12
	Turashvili	Ductal Breast Cell (10)	Invasive Lobular Breast Carcinoma (5)	19.676	3.648	.004
		Lobular Breast Cell (10)				
	Karnoub	Breast (15)	Invasive Ductal Breast Carcinoma (7)	10.640	4.160	2.49E–4
	Curtis	Breast (144)	Tubular Breast Carcinoma (67)	3.301	10.453	5.86E–19
	Richardson	Breast (7)	Ductal Breast Carcinoma (40)	4.822	6.049	3.40E–5
	Finak	Breast (6)	Invasive Breast Carcinoma (53)	**12.282**	15.498	6.91E–13
FNDC3A	Radvanyi	Breast (8)	Invasive Mixed Breast Carcinoma (3)	3.680	3.497	0.005
	Finak	Breast (6)	Invasive Breast Carcinoma (53)	2.047	17.398	4.36E–16
FNDC3B	Finak	Breast (6)	Invasive Breast Carcinoma (53)	3.980	10.909	1.11E–15
	TCGA	Breast (61)	Intraductal Cribriform Breast Adenocarcinoma (3)	–2.322	–18.767	8.26E–13
	Radvanyi	Breast (5)	Invasive Mixed Breast Carcinoma (3)	–3.307	–4.083	.008
	Gluck	Breast (4)	Invasive Breast Carcinoma (154)	–**2.106**	–6.251	.002
FNDC4	Turashvili	Ductal Breast Cell (10)	Invasive Ductal Breast Carcinoma (5)	–4.774	–3.595	.004
		Lobular Breast Cell (10)				
	TCGA	Breast (61)	Invasive Ductal Breast Carcinoma (389)	–**2.644**	–12.653	6.30E–22
		Breast (61)	Mixed Lobular and Ductal Breast Carcinoma (7)	–2.582	–5.422	2.64E–4
FNDC6	TCGA	Breast (61)	Intraductal Cribriform Breast Adenocarcinoma (3)	2.485	–7.213	3.22E–5
FNDC8	Radvanyi	Breast (7)	Ductal Breast Carcinoma in Situ (3)	–7.826	–6.295	.001
		Breast (7)	Invasive Mixed Breast Carcinoma (2)	–**2.437**	–4.100	.003
		Breast (7)	Invasive Lobular Breast Carcinoma (6)	–**2.468**	–2.889	.008
		Breast (7)	Invasive Ductal Breast Carcinoma (16)	–2.970	–3.532	.001

FNDC = fibronectin type-III domain-containing.

**Figure 2.**
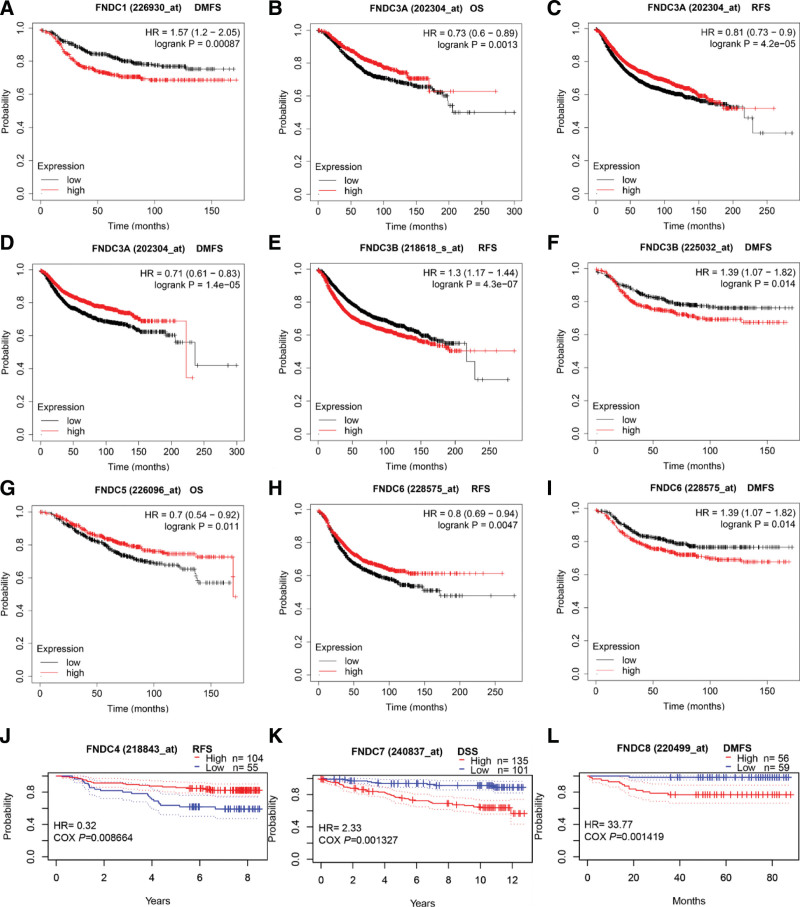
Survival curves of the FNDC family in breast cancer. (a-h): prognosis analysis of the FNDC family obtained from the KM plotter database. (i-j): prognosis analysis of FNDC4, FNDC7 and FNDC8 obtained from the KM plotter database, respectively. DMFS = distant metastasis free survival, DSS = disease specific survival, FNDC = fibronectin type-III domain-containing, KM = Kaplan-Meier, OS = overall survival, RFS = relapse free survival.

Next, we investigated whether the FNDC family is associated with the prognosis of breast cancer patients using the KM plotter and PrognoScan software programs. The OS, RFS, distant metastasis-free survival (DMFS), and post-progression survival (PPS) for each gene in the KM plotter database were investigated. All the significant results are shown in Figure 2. Increased FNDC1 (Fig. 2a), FNDC3B (Fig. 2e), FNDC7 (Fig. 2j), and FNDC8 (Fig. 2k) levels implied worse prognosis. In contrast, higher FNDC3A (Fig. 2b–d), FNDC4 (Fig. 2i), and FNDC5 (Fig. 2f) predicted better survival for breast cancer patients. Concerning FNDC6, There were 2 divergent consequences. Higher expression of FNDC6 was accompanied by longer RFS, whereas reduced FNDC6 expression revealed better DMFS. The results are presented in Table S1, Supplemental Digital Content, http://links.lww.com/MD/I34 of the supplementary file.

### 3.3. The expression level and prognostic value of the FNDC family in lung cancer

Similarly, taking advantage of the Oncomine database, we obtained relationships between FNDC family expression and lung cancer, which mainly includes lung adenocarcinoma and squamous cell lung carcinoma, but only FNDC1 and FNDC6 displayed significant results under the set thresholds. In light of Hou’s analysis, both FNDC1 and FNDC6 were upregulated in squamous cell lung carcinoma, and FNDC6 was significantly increased in lung adenocarcinoma. Details are presented in Table 2. Furthermore, we found that, with FNDC7 exception, expression of the FNDC family was much higher in lung cancer tissues than in normal tissues when we utilized TIMER, another online website, to compare tumor and normal expression (see Figure S1, Supplemental Digital Content, http://links.lww.com/MD/I31, which demonstrates the mRNA expression levels of the FNDC family in human cancers from TIMER).

**Table 2:** Datasets of FNDC family in lung cancer.

Gene	Dataset	Normal (cases)	Tumor (cases)	Fold change	*t* Test	*P* value
FNDC1	Hou	Lung (65)	Lung Adenocarcinoma (45)	4.910	9.551	1.33E–14
		Lung (65)	Squamous Cell Lung Carcinoma (27)	5.481	7.571	7.571
FNDC6	Hou	Lung (65)	Squamous Cell Lung Carcinoma (27)	6.792	7.900	1.03E–8

FNDC = fibronectin type-III domain-containing.

**Figure 3.**
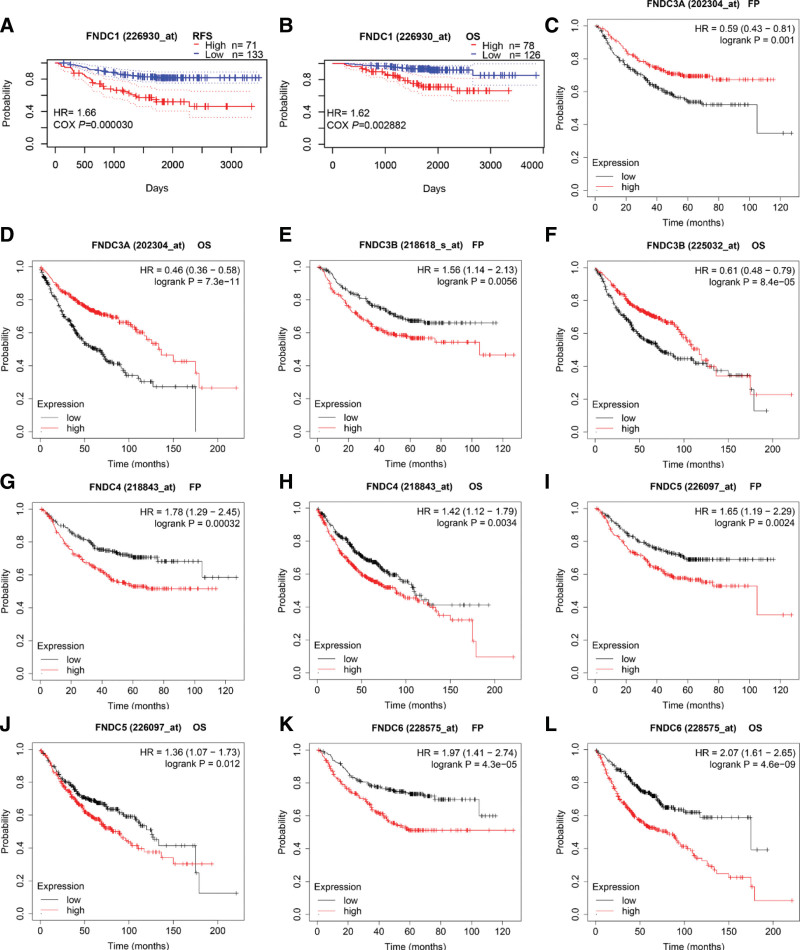
Survival curves of the FNDC family in lung adenocarcinoma. Survival analyses of FNDC3A (a, d), FNDC3B (e, f), FNDC4 (g, h), FNDC5 (i, j) and FNDC6 (k, l) were downloaded from the KM plotter database. Survival analyses of FNDC1 (b, c) were downloaded from the PrognoScan database. FNDC = fibronectin type-III domain-containing, FP = first progression, KM = Kaplan-Meier, OS = overall survival, RFS = relapse free survival.

The predictive relevance of the FNDC family in lung cancer was later demonstrated using the KM plotter and PrognoScan databases. Regarding lung cancer survival, the KM plotter examined the influence of the FNDC family on first progression (FP), OS, and PPS. Furthermore, RFS and OS were assessed for every gene using PrognoScan. Except for FNDC7 and FNDC8, each member of the FNDC family was correlated with statistically significant survival curves (Fig. 3). Unfortunately, no gene, with the exception of FNDC3A, was found to be statistically significant in patients with squamous cell lung cancer. In patients with lung adenocarcinoma, no genes from the FNDC family were shown to be associated with PPS, whereas all members were found to be associated with FP/RFS and OS. Higher FNDC3A expression is associated with better OS and FP rates (Fig. 3c and d). Conversely, upregulated FNDC1 (Fig. 3a and b), FNDC4 (Fig. 3g and h), FNDC5 (Fig. 3i and j), and FNDC6 (Fig. 3k and l) were associated with worse survival in patients with lung adenocarcinoma. It is worth noting that low and high expression level of FNDC3B was associated with better FP and OS, respectively (Fig. 3f and g). This may have been caused by the use of different probes. Table S2, Supplemental Digital Content, http://links.lww.com/MD/I35 in the supplementary file contains all specific prognostic analyses.

### 3.4. The expression level and prognostic value of the FNDC family in gastric cancer

On the 1 hand, there’re 3 different datasets for the FNDC family in gastric cancer in Oncomine. FNDC1 was shown to be significantly elevated in diffuse gastric adenocarcinoma, gastric intestinal type adenocarcinoma, gastric mixed adenocarcinoma, gastric adenocarcinoma and gastric cancer from Cho, Wang and DErrico datasets. However, the expression of FNDC3A, FNDC5, and FNDC8 was significantly reduced in gastric using the same 3 datasets. As shown in Table 3, the Oncomine database did not show statistically significant data for FNDC3B, FNDC4, FNDC6, or FNDC7. On the other hand, we found that each member of the FNDC family showed differential expression between stomach adenocarcinoma and normal tissue, as determined by TIMER (see Figure S1, Supplemental Digital Content, http://links.lww.com/MD/I31, which demonstrates the mRNA expression levels of the FNDC family in human cancers from TIMER).

**Table 3:** Datasets of FNDC family in gastric cancer.

Gene	Dataset	Normal (Cases)	Tumor (Cases)	Fold change	*t* Test	*P* value
FNDC1	Cho	Gastric Tissue (19)	Diffuse Gastric Adenocarcinoma (31)	7.225	8.778	2.21E–10
		Gastric Tissue (19)	Gastric Intestinal Type Adenocarcinoma (20)	5.194	5.969	3.85E–6
		Gastric Tissue (19)	Gastric Adenocarcinoma (4)	9.843	5.111	.007
		Gastric Tissue (19)	Gastric Mixed Adenocarcinoma (10)	2.619	3.480	.003
	Wang	Gastric Mucosa (12)	Gastric Cancer (12)	10.829	4.578	7.41E–5
		Gastric Tissue (3)				
	DErrico	Gastric Mucosa (31)	Gastric Intestinal Type Adenocarcinoma (26)	19.417	8.392	3.11E–11
		Gastric Mucosa (31)	Gastric Mixed Adenocarcinoma (4)	37.063	8.947	8.88E–5
		Gastric Mucosa (31)	Diffuse Gastric Adenocarcinoma (6)	10.855	4.104	.003
FNDC3A	DErrico	Gastric Mucosa (31)	Gastric Mixed Adenocarcinoma (4)	–2.485	–4.296	5.52E–4
FNDC5	DErrico	Gastric Mucosa (31)	Gastric Mixed Adenocarcinoma (4)	–4.378	–4.063	5.74E–4
		Gastric Mucosa (31)	Gastric Intestinal Type Adenocarcinoma (26)	–2.842	–3.285	9.66E–4
	Cho	Gastric Tissue (19)	Gastric Intestinal Type Adenocarcinoma (20)	–2.870	–4.250	9.88E–5
		Gastric Tissue (19)	Diffuse Gastric Adenocarcinoma (31)	–2.894	–4.614	5.55E–5
		Gastric Tissue (19)	Gastric Mixed Adenocarcinoma (10)	–2.718	–3.303	.002
FNDC8	DErrico	Gastric Mucosa (31)	Diffuse Gastric Adenocarcinoma (6)	–2.385	–3.366	.004

FNDC = fibronectin type-III domain-containing.

**Figure 4.**
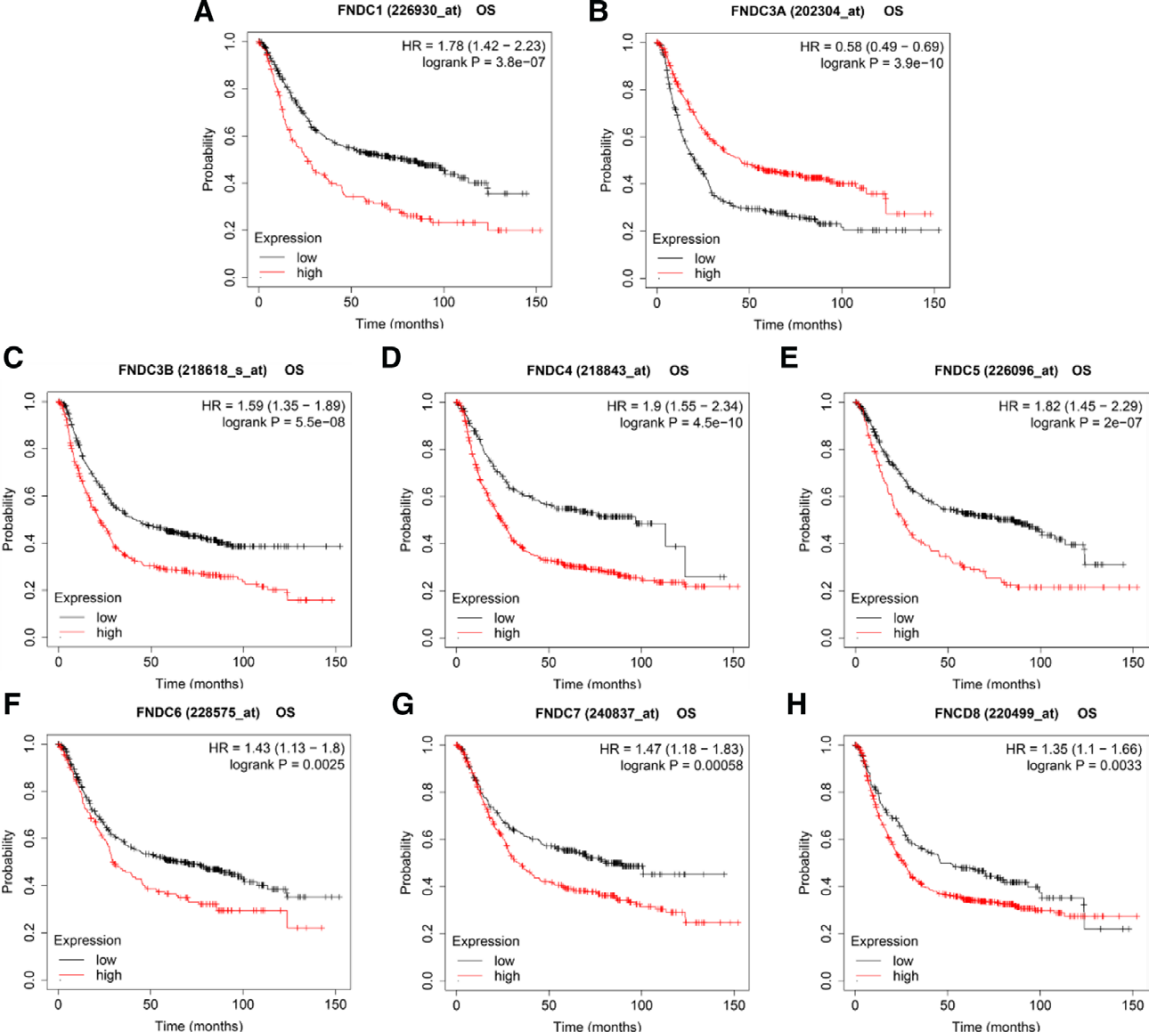
Survival curves of the FNDC family in gastric cancer. Survival analyses of FNDC1 (a), FNDC3A (b), FNDC3B (c), FNDC4 (d), FNDC5 (e), FNDC6 (f), FNDC7 (g) and FNDC8 (h) were obtained from the KM plotter database. FNDC = fibronectin type-III domain-containing, KM = Kaplan-Meier, OS = overall survival.

Next, we examined the relationships between members of the FNDC family and the survival results of patients with gastric cancer in the KM registry. This database provides OS, FP, and PPS data for the prognostic analyses of gastric cancer. Each gene of the FNDC family was closely related to OS, FP, and PPS, except for FNDC6, which was only associated with OS in patients with gastric cancer. Lower FNDC3A levels (Fig. 4b) indicated a worse prognosis, while low expression levels of other genes (Fig. 4a and 4c–h) in the FNDC family implied better OS. All the data are presented in Table S3, Supplemental Digital Content, http://links.lww.com/MD/I36 in the supplementary file.

### 3.5. The expression level and prognostic value of the FNDC family in liver cancer

**Figure 5.**
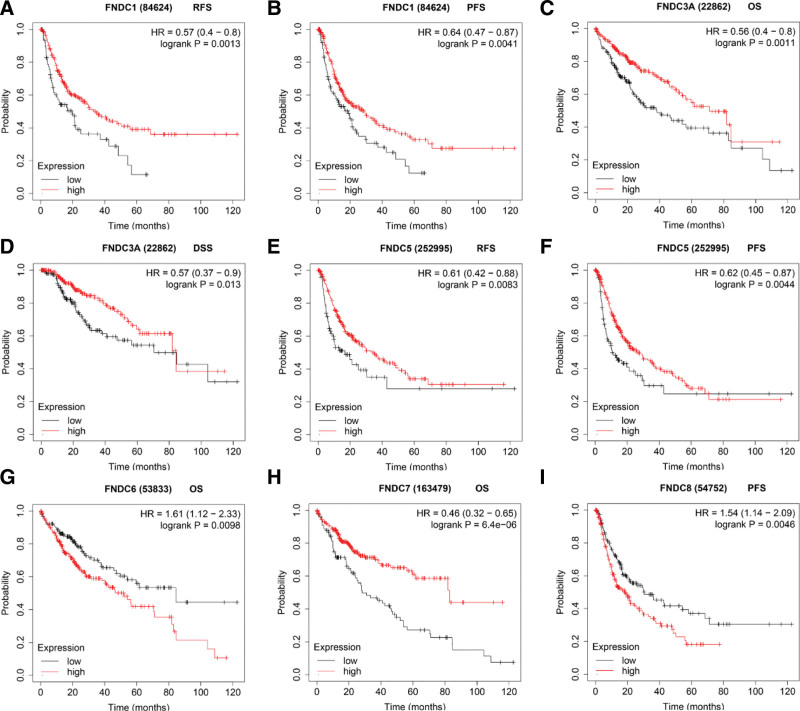
Survival curves of the FNDC family in liver cancer. Survival analyses of FNDC1 (a, b), FNDC3A (c, d), FNDC3B (e), FNDC5 (g, h), FNDC6 (i), FNDC7 (j) and FNDC8 (k) were obtained from the KM plotter database. DSS = disease specific survival, FNDC = fibronectin type-III domain-containing, KM = Kaplan-Meier, OS = overall survival, PFS = progression free survival, RFS = relapse free survival.

Only 4 out of 10 analyses met our threshold for FNDC4 in 3 out of 7 datasets from the Oncomine database, and all analyses showed low expression of FNDC4. Furthermore, we obtained the same results for FNDC4 expression in liver cancer from TIMER (see Figure S1 d, Supplemental Digital Content, http://links.lww.com/MD/I31, which demonstrates the mRNA expression levels of the FNDC family in human cancers from TIMER). Additionally, compared to normal liver tissues, FNDC3A and FNDC4 showed lower expression, while FNDC3B and FNDC8 displayed higher expression in liver cancer. Significant statistical differences in the prognostic results of the FNDC family in liver cancer were observed only in the KM plotter. OS, RFS, progression-free survival (PFS), and disease-specific survival (DSS) were analyzed in patients with liver cancer using a KM plotter. With respect to liver cancer, high expression of FNDC1 (Fig. 5a and b), FNDC3A (Fig. 5c and d), FNDC5 (Fig. 5e and f), and FNDC7 (Fig. 5h) was correlated with better prognosis, but increased expression of FNDC6 (Fig. 5g) and FNDC8 (Fig. 5i) was correlated with poor OS and PFS, respectively. Table 4 shows all detailed datasets from the Oncomine database. All the data are presented in Table S5, Supplemental Digital Content, http://links.lww.com/MD/I38 in the supplementary file.

**Table 4:** Datasets of FNDC family in liver cancer.

Gene	Dataset	Normal (Cases)	Tumor (Cases)	Fold change	*t* Test	*P* value
FNDC4	Mas	Liver (19)	Hepatocellular Carcinoma (38)	–2.664	–11.532	2.68E–16
		Liver (19)	Cirrhosis (58)	–2.097	–10.242	1.49E–13
	Roessler	Liver (21)	Hepatocellular Carcinoma (22)	–2.116	–5.764	4.82E–7
	Wurmbach	Liver (10)	Hepatocellular Carcinoma (35)	–3.723	–4.771	1.01E–4

FNDC = fibronectin type-III domain-containing.

### 3.6. The expression level and prognostic value of the FNDC family in colorectal cancer

**Figure 6.**
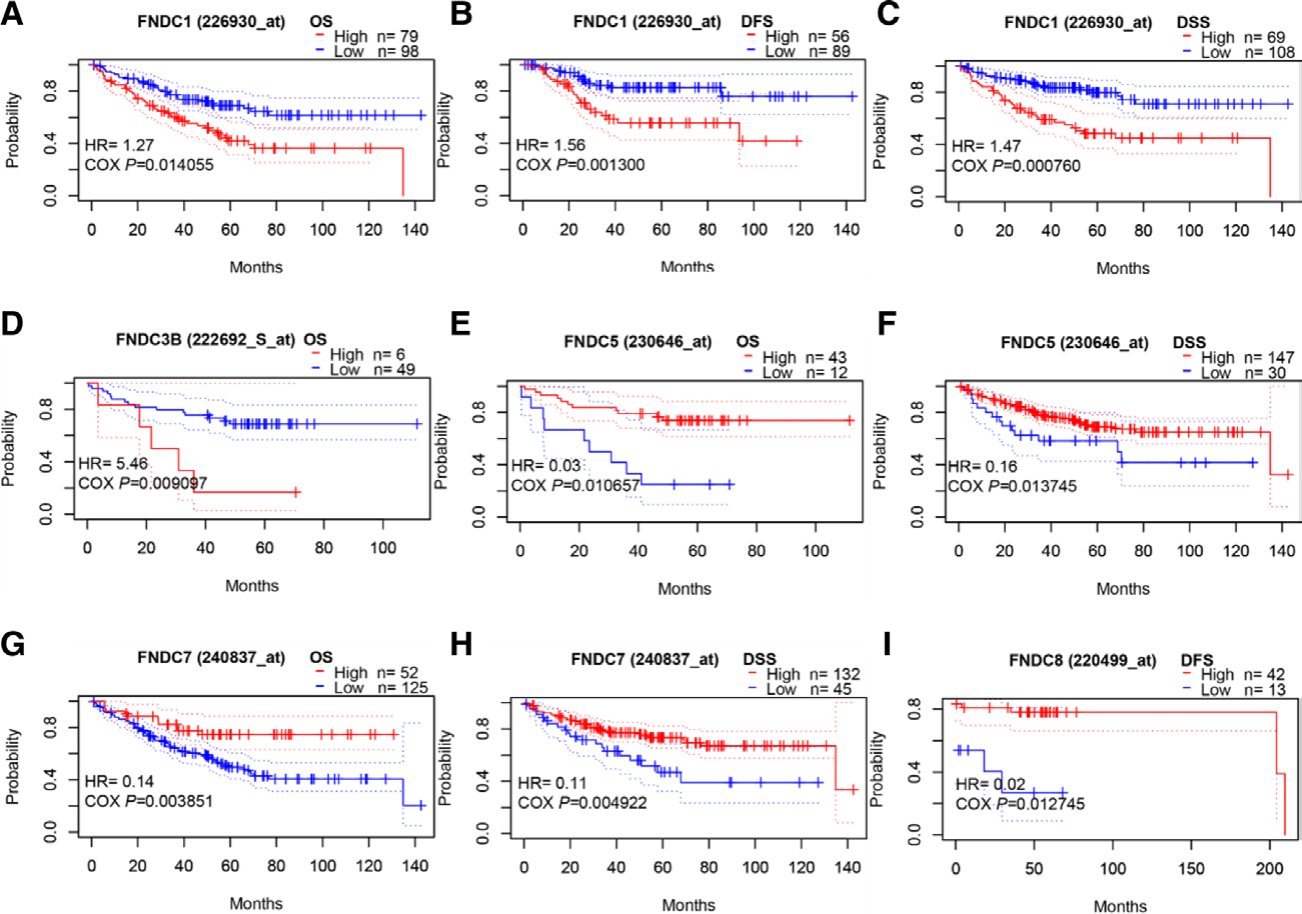
Survival curves of the FNDC family in ovarian cancer. Survival analyses of FNDC1 (a, b), FNDC3A (c, d), FNDC3B (e), FNDC5 (g, h), FNDC6 (i, j), FNDC7 (k, l) and FNDC8 (m, n) were obtained from the KM plotter database. Survival analysis of FNDC4 (f) was obtained from the PrognoScan database. FNDC = fibronectin type-III domain-containing, KM = Kaplan-Meier, OS = overall survival, PFS, progression free survival.

**Figure 7.**
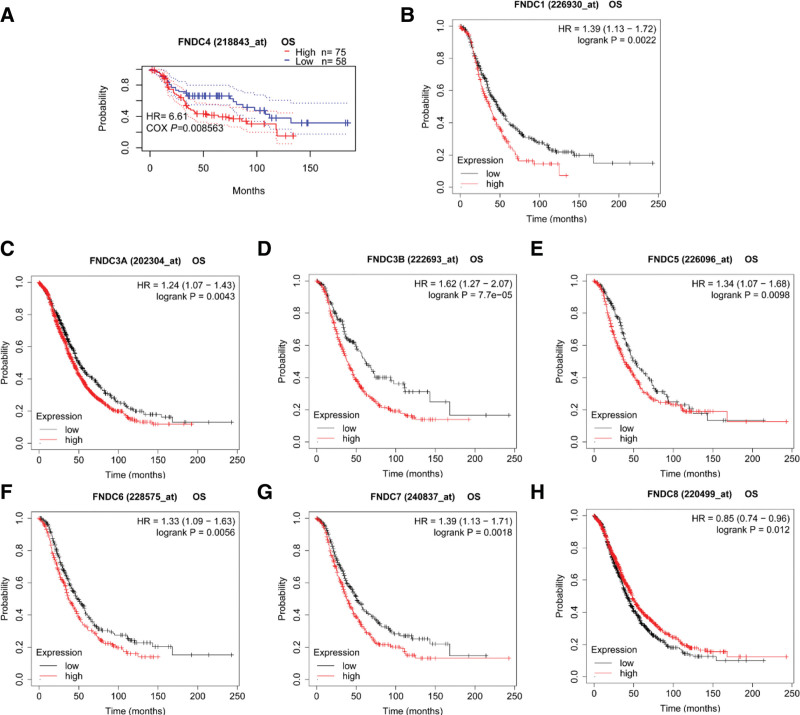
Survival curves of the FNDC family in colorectal cancer. Survival analyses of FNDC1 (a, b, c), FNDC3B (d), FNDC5 (e, f), FNDC7 (g, h) and FNDC8 (i) were obtained from the KM plotter database. DFS = disease free survival, DSS = disease specific survival, FNDC = fibronectin type-III domain-containing, OS = overall survival.

In comparison to normal tissues, FNDC1 and FNDC3B were highly expressed in colorectal cancer using the Oncomine database. Moreover, low expression of FNDC5 and high expression of FNDC8 were observed in colon adenocarcinoma according to the TIMER database (see Figure S1 e, Supplemental Digital Content, http://links.lww.com/MD/I31) which demonstrates the mRNA expression levels of the FNDC family in human cancers from TIMER). We then took advantage of the PrognoScan database to study the prognostic value of the FNDC family in colorectal cancer. As shown in Figure 6, OS, DFS, and DSS survival curves of patients with low FNDC1 expression were associated with the long survival time of colorectal cancer patients (Figure 7a–c). Consistently, decreased FNDC3B expression was associated with good OS (Fig. 7d). Nevertheless, higher FNDC5 (Fig. 7e and f) and FNDC7 (Fig. 7g and h) showed better OS and DSS, and reduced FNDC8 (Fig. 7i) predicted worse DSS. All the data are presented in Table S6, Supplemental Digital Content, http://links.lww.com/MD/I39 in the supplementary file.

### 3.7. The expression level and prognostic value of the FNDC family in ovarian cancer

In the Oncomine database, there are only 3 datasets for FNDC3A, FNDC4, and FNDC5, which indicate statistically notable distinctions between prostate tumors and normal tissues. In accordance with Bonome analysis, FNDC3A was downregulated in ovarian carcinoma. Meanwhile, the expression levels of FNDC4 and FNDC5 were lower in ovarian serous adenocarcinoma in the Yoshihara dataset. In addition, we observed the expression of the FNDC family members, except FNDC7, in ovarian serous cystadenocarcinoma, the most frequent type of ovarian cancer, using TIMER (see Figure S1, Supplemental Digital Content, http://links.lww.com/MD/I31, which demonstrates the mRNA expression levels of the FNDC family in human cancers from TIMER). Table 5 shows all detailed datasets from the Oncomine database.

**Table 5:** Datasets of FNDC family in ovarian cancer.

Gene	Dataset	Normal (Cases)	Tumor (Cases)	Fold change	*t* Test	*P* value
FNDC3A	Bonome	Ovarian Surface Epithelium (10)	Ovarian Carcinoma (185)	–3.084	–14.597	6.96E–10
FNDC4	Yoshihara	Peritoneum (10)	Ovarian Serous Adenocarcinoma (42)	–6.531	–6.750	2.80E–7
FNDC5	Yoshihara	Peritoneum (10)	Ovarian Serous Adenocarcinoma (40)	–6.535	–3.532	7.68E–9

FNDC = fibronectin type-III domain-containing.

Next, we examined how the FNDC family was linked to ovarian cancer patient survival outcomes using the KM plotter and PrognoScan databases. Using the KM plotter database, we analyzed the survival outcomes of ovarian cancer patients, including PFS, OS, and PPS. In addition, the PrognoScan database offers significant prognostic results for patients with ovarian cancer. As shown in Figure 6, all FNDCs have a strong connection to the OS (Fig. 6a–g). Except for FNDC8, high expression of the FNDC family members was associated with poor prognosis in ovarian cancer patients (Fig. 6h). All the data are presented in Table S4, Supplemental Digital Content, http://links.lww.com/MD/I37 in the supplementary file.

## 4. Discussion

Through the use of the Oncomine and TIMER databases, we conducted a thorough examination of the mRNA expression levels of the FNDC family members across a wide range of cancer types in the current study. We focused on the most prevalent types of cancer, such as breast, lung, gastric, ovarian, liver, and colorectal cancers, as well as the most common subtypes of each cancer type. As an additional search strategy, we examined the predictive significance of this family in patients with cancer using the KM plotter and PrognoScan websites.

It is worth mentioning that the transcriptional expression levels in certain additional solid tumors, such as esophageal, kidney, leukemia, lymphoma, brain, and central nervous system malignancies, exhibited substantial differences (Fig. 1). To that end, we probed the predictive value of the FNDC family in all cancers previously mentioned in the PrognoScan database and found that the results in Table S7, Supplemental Digital Content, http://links.lww.com/MD/I40 in the supplemental file were statistically significant. In summary, there were no significant predictive effects of FNDC family members in any of the malignancies studied, except for blood cancer. In addition, some members of the FNDC family have shown significant prognostic outcomes in bladder and skin cancers. As shown in Figure S2, Supplemental Digital Content, http://links.lww.com/MD/I32, higher FNDC1, FNDC5, and FNDC6 levels predicted worse DSS in bladder cancer. Moreover, the expression of FNDC3A, FNDC3B, FNDC5, and FNDC6 has been implicated in the prognosis of blood cancers. Furthermore, the poor prognosis of skin cancer patients was significantly associated with increased FNDC3A (see Figure S2, Supplemental Digital Content, http://links.lww.com/MD/I32, which demonstrates survival curves of the FNDC family in bladder, blood and skin cancers via the PrognoScan database).

Patients with stomach, breast, and prostate malignancies have frequently been found to have FNDC1 upregulation in clinical samples,^[3,17,31]^ and FNDC3B is increased in liver, lung, and colorectal cancers.^[5,32,33]^ Our expression analyses revealed that the mRNA levels of FNDC1 and FNDC3B were significantly higher in most malignancies than in normal tissues, which is consistent with previous studies. In terms of FNDC3A, there are only a few recent studies on its significance to cancer, however, it has been discovered to be strongly expressed in colorectal and cervical cancers, among other malignancies.^[4,19]^ In our study, we found that FNDC3A was more common in most types of cancers. For FNDC4, differential expression was not detected in colorectal cancer tissues but increased in inflammatory bowel disease samples.^[4]^ Similar to previously reported results in other studies, the majority of malignancies in our analysis had decreased mRNA expression. Contrarily, FNDC5 has been found to be highly expressed in a variety of cancerous tissues and cells, including breast and kidney cancers.^[34,35]^ Different detection methods, sample sources, and histological types may have led to contradictory results. Besides, we noticed that the transcriptional expression levels of FNDC6 varied depending on the tumor type, with lower levels in esophageal, breast, and prostate cancers and higher levels in lung and pancreatic cancers being found in some cases. There were few datasets for FNDC7 and FNDC8 in our analyses, and they have rarely been reported so far.

Four distinct biochemical subgroups of breast cancer have emerged in recent years through comprehensive transcriptional profiling research: luminal A (ER+/HER2-/grade 1 or 2), luminal B (ER+/HER2-/grade 3), HER2 (HER2 + tumor) enriched, and basal-like (ER-/PR-/HER2-). These subgroups have been demonstrated to be consistent in their ability to predict therapy sensitivity and survival results.^[36]^ Thus, we used the KM Plotter to examine prognostic outcomes based on the 4 intrinsic categories of disease.^[37]^ All data are shown in Table S8, Supplemental Digital Content, http://links.lww.com/MD/I41 in the supplementary file, and all statistically significant survival curves are displayed in Figure S3, Supplemental Digital Content, http://links.lww.com/MD/I33. Furthermore, decreased FNDC3Awas found to be related with a poor RFS or OS in all subtypes of patients apart from luminal B. It’s worth noting that high FNDC3B expression was probably tied to completely opposite survival outcomes in different breast cancer subtypes and survival analysis methods. For example, in patients with luminal A classification, lower FNDC3B levels were accompanied by worse RFS, while better OS and PPS were related to decreased FNDC3B levels in patients with the same subtype. In addition, only 1 significant prognostic analysis of FNDC4 was found in the 4 intrinsic subtypes of breast cancer. Additionally, higher FNDC5 levels predicted better RFS and DMFS in basal patients. Moreover, increased FNDC6 was associated with good RFS in luminal B and basal patients, but poor PPS with high FNDC6 was detected in basal patients. Analyses of the FNDC family in breast cancer have already shown that increased FNDC3A or FNDC5 indicated good survival results (see Figure S3, Supplemental Digital Content, http://links.lww.com/MD/I33, which demonstrates the survival curves of the FNDC family in different St Gallen subtypes of breast cancer). As a result, we propose that FNDC3A could be used as a predictive biomarker in the treatment of breast cancer, particularly in basal and luminal A subtypes. Additionally, FNDC5 may serve as a prognostic biomarker for basal breast cancer.

Lung cancer is the main cause of cancer-related death in China and around the globe.^[38]^ Lung cancer includes 2 subtypes: squamous cell lung carcinoma and lung adenocarcinoma. Our prognostic studies revealed that no gene was linked with OS or PPS in patients with squamous cell lung cancer, nor was any gene associated with PPS in patients with lung adenocarcinoma. However, in lung adenocarcinoma, all members of the FNDC family, except FNDC7 and FNDC8, are interrelated to RFS/OS and FP. This leads us to believe that, for lung adenocarcinoma, this set of genes can be used as a biomarker to predict prognosis, rather than squamous cell lung cancer, as previously thought.

Furthermore, we found that members of the FNDC family could be potential therapeutic targets or prognostic markers in several cancers, in line with earlier reports.^[39,40]^ What’s more, we had a few other new findings that upregulated FNDC3A mRNA expression signified better OS in gastric and liver cancer patients. FNDC1 High expression was tightly correlated with poor prognosis in ovarian cancer but with good RFS and PFS in liver cancer. A potential interaction between FNDC3B and survival outcomes has also been observed in ovarian and gastric cancer. FNDC4 and FNDC6 are relevant to survival curves in colorectal and breast cancer. Regretfully, it seems that no significant survival results were related to the FNDC family in brain cancer.

Our research will help us to gain a better understanding of the FNDC family’s expression levels and prognostic usefulness in a few solid cancers. Moreover, our findings provide evidence that members of this family can be used as new prognostic biomarkers or prospective targets for cancer therapy in humans. However, we only focused on the mRNA expression level of FNDCs and its prognostic value and did not analyze its protein expression levels and some probable signal routings. Sample cohort studies are required to confirm the prognostic value of this family, and additional studies are required to identify the molecular pathways that underlie the development of malignant malignancies. In summary, we conducted a systematic analysis of not only the mRNA expression levels but also the prognostic value of the FNDC family in a spectrum of typical malignancies, including breast, lung, gastric, ovarian, liver, and colorectal cancers. Multiple members, including FNDC1, FNDC3A, and FNDC3B, demonstrated significant expression changes between cancer and surrounding normal tissue groups in the malignancies studied in this study. Furthermore, we developed a new biomarker, FNDC3A, for the prognosis of breast cancer, and lung adenocarcinoma might be able to choose the FNDC family as a prognostic biomarker.

## Acknowledgments

We are grateful to the contributors of the data to Oncomine, TIMER, Kaplan–Meier plotter, and PrognScan.

## Author contributions

**Conceptualization:** Hui Jiang.

**Data curation:** Hui Jiang.

**Resources:** Zhi Liu.

**Validation:** Bo Ling Chu.

**Writing – original draft:** Bo Ling Chu, Jiao He.

**Writing – review & editing:** Ling Yang.

## Supplementary Material



## References

[1] Berraondo P Labiano S Minute L. Cellular immunotherapies for cancer. Oncoimmunology. 2017;6:e1306619. 28638729 10.1080/2162402X.2017.1306619 PMC5467985

[2] Rhodes DR Kalyana-Sundaram S Mahavisno V. Oncomine 3.0: genes, pathways, and networks in a collection of 18,000 cancer gene expression profiles. Neoplasia. 2007;9:166–80. 17356713 10.1593/neo.07112 PMC1813932

[3] Ren J Niu G Wang X. Overexpression of FNDC1 in gastric cancer and its prognostic significance. J Cancer. 2018;9:4586–95. 30588242 10.7150/jca.27672 PMC6299387

[4] Wuensch T Wizenty J Quint J. Expression analysis of fibronectin type III domain-containing (FNDC) genes in inflammatory bowel disease and colorectal cancer. Gastroenterol Res Pract. 2019;2019:3784172. 31093274 10.1155/2019/3784172 PMC6481110

[5] Lin CH Lin YW Chen YC. FNDC3B promotes cell migration and tumor metastasis in hepatocellular carcinoma. Oncotarget. 2016;7:49498–508. 27385217 10.18632/oncotarget.10374 PMC5226524

[6] Das DK Naidoo M Ilboudo A. miR-1207-3p regulates the androgen receptor in prostate cancer via FNDC1/fibronectin. Exp Cell Res. 2016;348:190–200. 27693493 10.1016/j.yexcr.2016.09.021 PMC5077722

[7] Fan X Chen X Deng W. Up-regulated microRNA-143 in cancer stem cells differentiation promotes prostate cancer cells metastasis by modulating FNDC3B expression. BMC Cancer. 2013;13:61. 23383988 10.1186/1471-2407-13-61 PMC3585861

[8] Xu H Hu Y Qiu W. Potential mechanisms of microRNA-129-5p in inhibiting cell processes including viability, proliferation, migration and invasiveness of glioblastoma cells U87 through targeting FNDC3B. Biomed Pharmacother. 2017;87:405–11. 28068630 10.1016/j.biopha.2016.12.100

[9] Zeng W Liu Y Li WT. CircFNDC3B sequestrates miR-937-5p to derepress TIMP3 and inhibit colorectal cancer progression. Mol Oncol. 2020;14:2960–84. 32896063 10.1002/1878-0261.12796 PMC7607164

[10] Kishimoto K Kato A Osada S. Fad104, a positive regulator of adipogenesis, negatively regulates osteoblast differentiation. Biochem Biophys Res Commun. 2010;397:187–91. 20493170 10.1016/j.bbrc.2010.05.077

[11] Bosma M Gerling M Pasto J. FNDC4 acts as an anti-inflammatory factor on macrophages and improves colitis in mice. Nat Commun. 2016;7:11314. 27066907 10.1038/ncomms11314 PMC4832079

[12] Kim HK Jeong YJ Song IS. Glucocorticoid receptor positively regulates transcription of FNDC5 in the liver. Sci Rep. 2017;7:43296. 28240298 10.1038/srep43296 PMC5327437

[13] Hayashi H Al Mamun A Sakima M. Activator of G-protein signaling 8 is involved in VEGF-mediated signal processing during angiogenesis. J Cell Sci. 2016;129:1210–22. 26826188 10.1242/jcs.181883

[14] Sato M Jiao Q Honda T. Activator of G protein signaling 8 (AGS8) is required for hypoxia-induced apoptosis of cardiomyocytes: role of G betagamma and connexin 43 (CX43). J Biol Chem. 2009;284:31431–40. 19723622 10.1074/jbc.M109.014068 PMC2781539

[15] Deng AY Chauvet C Menard A. Alterations in fibronectin type III domain containing 1 protein gene are associated with hypertension. PLoS One. 2016;11:e0151399. 27064407 10.1371/journal.pone.0151399 PMC4827815

[16] Jiang T Gao W Lin S. FNDC1 promotes the invasiveness of gastric cancer via Wnt/beta-catenin signaling pathway and correlates with peritoneal metastasis and prognosis. Front Oncol. 2020;10:590492. 33392086 10.3389/fonc.2020.590492 PMC7773909

[17] Yunwen C Shanshan G Zhifei B. The silencing of FNDC1 inhibits the tumorigenesis of breast cancer cells via modulation of the PI3K/Akt signaling pathway. Mol Med Rep. 2021;23:479. 33899120 10.3892/mmr.2021.12118 PMC8097762

[18] Tan M Schaffalitzky de Muckadell OB Joergensen MT. Gene expression network analysis of precursor lesions in familial pancreatic cancer. J Pancreat Cancer. 2020;6:73–84. 32783019 10.1089/pancan.2020.0007 PMC7415888

[19] Yu J Liang LL Liu J. Development and validation of a novel gene signature for predicting the prognosis by identifying m5C modification subtypes of cervical cancer. Front Genet. 2021;12:733715. 34630524 10.3389/fgene.2021.733715 PMC8493221

[20] Cheng CK Wang AZ Wong THY. FNDC3B is another novel partner fused to RARA in the t(3;17)(q26;q21) variant of acute promyelocytic leukemia. Blood. 2017;129:2705–9. 28314734 10.1182/blood-2017-02-767707

[21] Zhong Z Zhang H Hong M. FNDC3B promotes epithelial-mesenchymal transition in tongue squamous cell carcinoma cells in a hypoxic microenvironment. Oncol Rep. 2018;39:1853–9. 29393475 10.3892/or.2018.6231

[22] Wang B Zheng B Lu Y. FNDC4 acts as an extracellular factor to promote the invasiveness of hepatocellular carcinoma partly via the PI3K/Akt signalling pathway. Cancer Med. 2021;10:7242–52. 34418326 10.1002/cam4.4225 PMC8525097

[23] Bostrom P Wu J Jedrychowski MP. A PGC1-alpha-dependent myokine that drives brown-fat-like development of white fat and thermogenesis. Nature. 2012;481:463–8. 22237023 10.1038/nature10777 PMC3522098

[24] Cui XF Cui XG Leng N. Overexpression of interleukin-20 receptor subunit beta (IL20RB) correlates with cell proliferation, invasion and migration enhancement and poor prognosis in papillary renal cell carcinoma. J Toxicol Pathol. 2019;32:245–51. 31719751 10.1293/tox.2019-0017 PMC6831501

[25] Wu C Li M Meng H. Analysis of status and countermeasures of cancer incidence and mortality in China. Sci China Life Sci. 2019;62:640–7. 30900169 10.1007/s11427-018-9461-5

[26] Li T Fu J Zeng Z. TIMER2.0 for analysis of tumor-infiltrating immune cells. Nucleic Acids Res. 2020;48:W509–14. 32442275 10.1093/nar/gkaa407 PMC7319575

[27] Lanczky A Nagy A Bottai G. miRpower: a web-tool to validate survival-associated miRNAs utilizing expression data from 2178 breast cancer patients. Breast Cancer Res Treat. 2016;160:439–46. 27744485 10.1007/s10549-016-4013-7

[28] Mizuno H Kitada K Nakai K. PrognoScan: a new database for meta-analysis of the prognostic value of genes. BMC Med Genomics. 2009;2:18. 19393097 10.1186/1755-8794-2-18 PMC2689870

[29] Zheng RS Sun KX Zhang SW. [Report of cancer epidemiology in China, 2015]. Zhonghua Zhong Liu Za Zhi. 2019;41:19–28. 30678413 10.3760/cma.j.issn.0253-3766.2019.01.005

[30] Feng RM Zong YN Cao SM. Current cancer situation in China: good or bad news from the 2018 global cancer statistics? Cancer Commun (Lond). 2019;39:22. 31030667 10.1186/s40880-019-0368-6 PMC6487510

[31] Das DK Ogunwobi OO. A novel microRNA-1207-3p/FNDC1/FN1/AR regulatory pathway in prostate cancer. RNA Dis. 2017;4:e1503. 28251177 PMC5328418

[32] Bian T Zheng L Jiang D. Overexpression of fibronectin type III domain containing 3B is correlated with epithelial-mesenchymal transition and predicts poor prognosis in lung adenocarcinoma. Exp Ther Med. 2019;17:3317–26. 30988707 10.3892/etm.2019.7370 PMC6447801

[33] Li Y Yang J Wang H. FNDC3B, targeted by miR-125a-5p and miR-217, promotes the proliferation and invasion of colorectal cancer cells via PI3K/mTOR signaling. Onco Targets Ther. 2020;13:3501–10. 32431508 10.2147/OTT.S226520 PMC7201223

[34] Tejeda ME Canto P Tenorio-Torres A. Increased FNDC5/IRISIN protein expression in breast cancer tissue is associated with obesity in postmenopausal women. J Clin Pathol. 2021:jclinpath-2020-207249. 10.1136/jclinpath-2020-207249 34083413

[35] Altay DU Keha EE Karaguzel E. The diagnostic value of FNDC5/Irisin in renal cell cancer. Int Braz J Urol. 2018;44:734–9. 29522296 10.1590/S1677-5538.IBJU.2017.0404 PMC6092672

[36] Perou CM Sorlie T Eisen MB. Molecular portraits of human breast tumours. Nature. 2000;406:747–52. 10963602 10.1038/35021093

[37] Gyorffy B Lanczky A Eklund AC. An online survival analysis tool to rapidly assess the effect of 22,277 genes on breast cancer prognosis using microarray data of 1,809 patients. Breast Cancer Res Treat. 2010;123:725–31. 20020197 10.1007/s10549-009-0674-9

[38] Chen W Zheng R Baade PD. Cancer statistics in China, 2015. CA Cancer J Clin. 2016;66:115–32. 26808342 10.3322/caac.21338

[39] Zhong M Zhang Y Yuan F. High FNDC1 expression correlates with poor prognosis in gastric cancer. Exp Ther Med. 2018;16:3847–54. 30402143 10.3892/etm.2018.6731 PMC6201048

[40] Han B Wang H Zhang J. FNDC3B is associated with ER stress and poor prognosis in cervical cancer. Oncol Lett. 2020;19:406–14. 31897153 10.3892/ol.2019.11098 PMC6924122

