# Supplementary material for: Expression and prognosis analyses of the fibronectin type-III domain-containing (FNDC) protein family in human cancers: A Review
Source: Medicine (Baltimore). 2022 Dec 9;101(49):e31854. doi: 10.1097/MD.0000000000031854 (PMC9750624; doi:10.1097/MD.0000000000031854)
Supplement: Supplementary file 1 [file medi-101-e31854-s001.pdf]

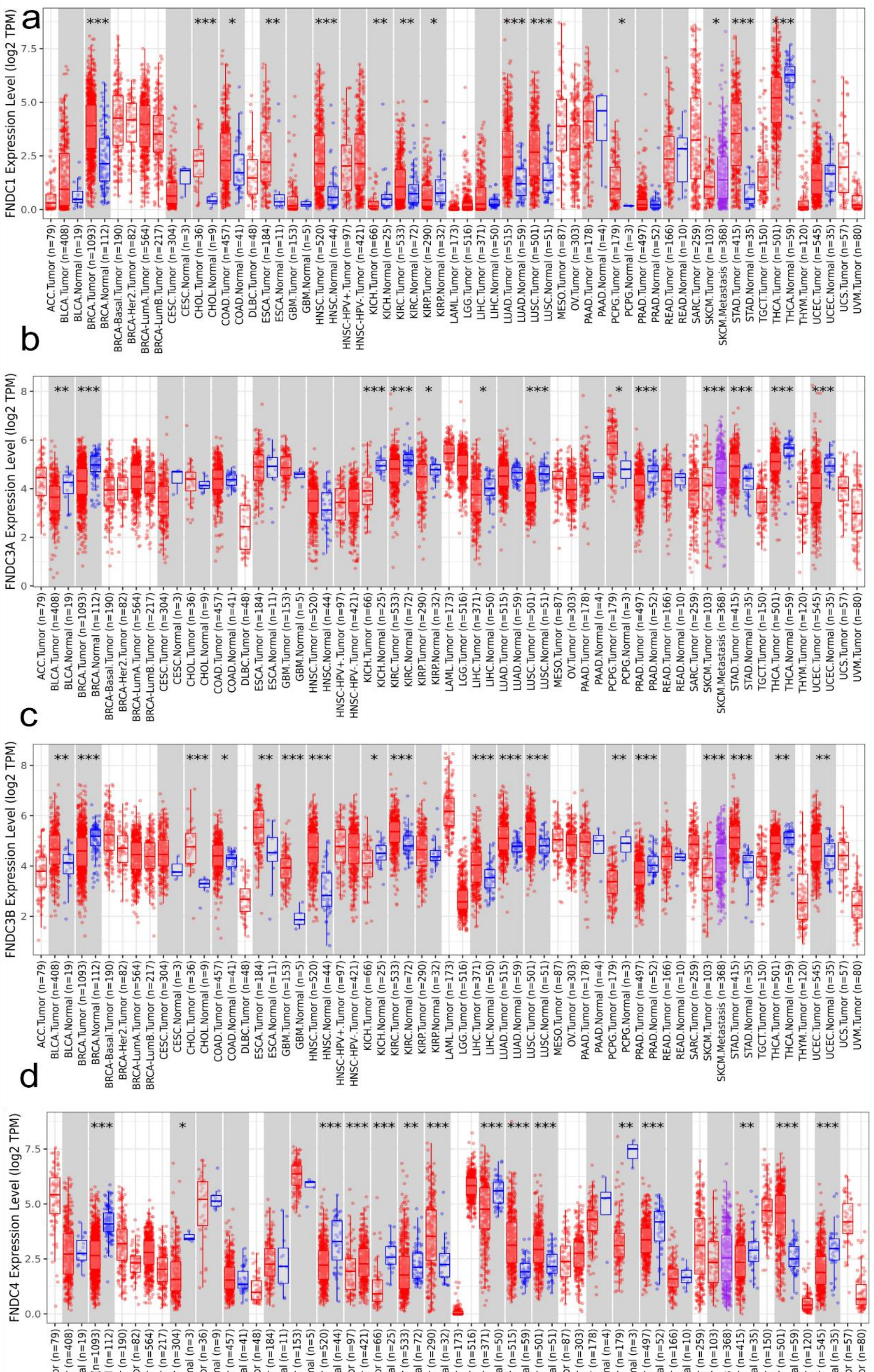
a
FNDC1 Expression Level (log2 TPM)
b
FNDC3A Expression Level (log2 TPM)
c
FNDC3B Expression Level (log2 TPM)
d
FNDC4 Expression Level (log2 TPM)

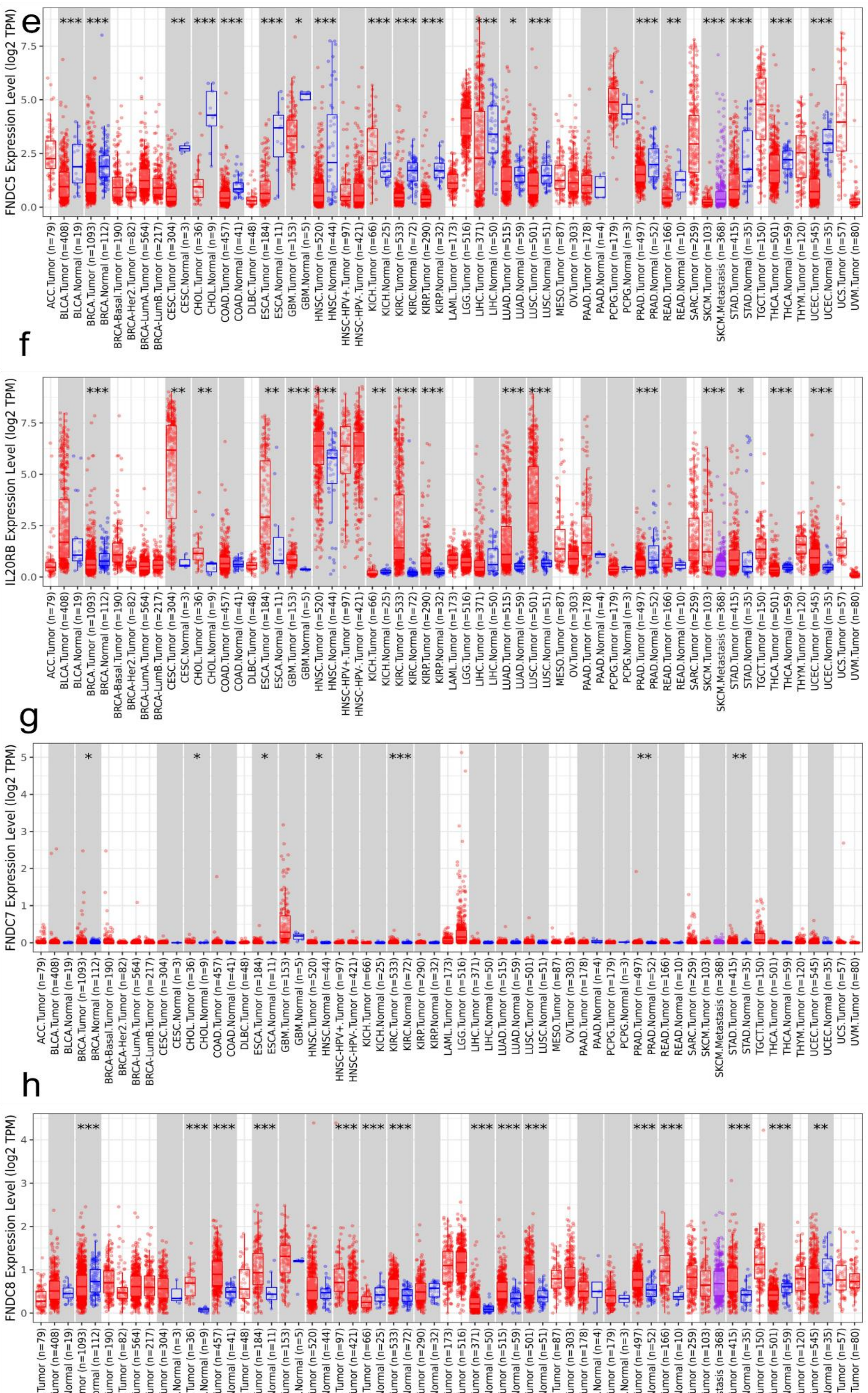
e
FNDC5 Expression Level (log2 TPM)
f
IL20RB Expression Level (log2 TPM)
g
FNDC7 Expression Level (log2 TPM)
h
FNDC8 Expression Level (log2 TPM)
ACC.Tumor (n=79)
BLCA.Tumor (n=408)
BLCA.Normal (n=19)
BRCA.Tumor (n=1093)
BRCA.Normal (n=112)
BRCA-Basal.Tumor (n=190)
BRCA-Her2.Tumor (n=82)
BRCA-LumA.Tumor (n=564)
BRCA-LumB.Tumor (n=217)
CESC.Tumor (n=304)
CESC.Normal (n=3)
CHOL.Tumor (n=36)
CHOL.Normal (n=9)
COAD.Tumor (n=457)
COAD.Normal (n=41)
DLBC.Tumor (n=48)
ESCA.Tumor (n=184)
ESCA.Normal (n=11)
GBM.Tumor (n=153)
GBM.Normal (n=5)
HNSC.Tumor (n=520)
HNSC.Normal (n=44)
HNSC-HPV+.Tumor (n=97)
HNSC-HPV-.Tumor (n=421)
KICH.Tumor (n=66)
KICH.Normal (n=25)
KIRC.Tumor (n=533)
KIRC.Normal (n=72)
KIRP.Tumor (n=290)
KIRP.Normal (n=32)
LAML.Tumor (n=173)
LGG.Tumor (n=516)
LIHC.Tumor (n=371)
LIHC.Normal (n=50)
LUAD.Tumor (n=515)
LUAD.Normal (n=59)
LUSC.Tumor (n=501)
LUSC.Normal (n=51)
MESO.Tumor (n=87)
OV.Tumor (n=303)
PAAD.Tumor (n=178)
PAAD.Normal (n=4)
PCPG.Tumor (n=179)
PCPG.Normal (n=3)
PRAD.Tumor (n=497)
PRAD.Normal (n=52)
READ.Tumor (n=166)
READ.Normal (n=10)
SARC.Tumor (n=259)
SKCM.Tumor (n=103)
SKCM.Metastasis (n=368)
STAD.Tumor (n=415)
STAD.Normal (n=35)
TGCT.Tumor (n=150)
THCA.Tumor (n=501)
THCA.Normal (n=59)
THYM.Tumor (n=120)
UCEC.Tumor (n=545)
UCEC.Normal (n=35)
UCS.Tumor (n=57)
UVM.Tumor (n=80)

Supplementary Fig 1. The mRNA expression levels of the FNDC family in human cancers from TIMER. The differential expressions of FNDC1 (a), FNDC3A (b), FNDC3B (c), FNDC4 (d), FNDC5 (e), FNDC6/IL20RB (f), FNDC7 (g) and FNDC8 (h) were checked using the RNA-seq data of multiple malignancies in TCGA.
