# Supplementary material for: Expression and prognosis analyses of the fibronectin type-III domain-containing (FNDC) protein family in human cancers: A Review
Source: Medicine (Baltimore). 2022 Dec 9;101(49):e31854. doi: 10.1097/MD.0000000000031854 (PMC9750624; doi:10.1097/MD.0000000000031854)
Supplement: Supplementary file 2 [file medi-101-e31854-s002.pdf]

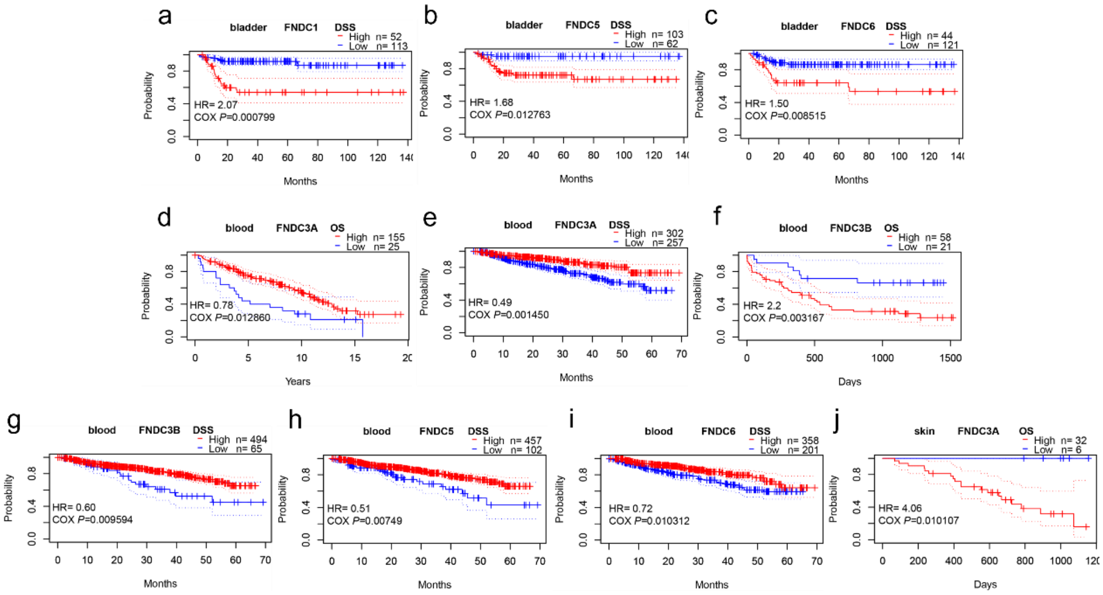

Supplementary Fig 2. Survival curves of the FNDC family in bladder, blood and skin cancers via the PrognoScan database. Survival curves of FNDC1 (a), FNDC5 (b) and FNDC6 (c) in bladder cancer. Survival curves of FNDC3A (d, e), FNDC3B (f, g), FNDC5 (h) and FNDC6 (i) in blood cancer. Survival curves of FNDC3A (j) in skin cancer. OS, overall survival; DSS, disease specific survival.
