# Supplementary material for: Expression and prognosis analyses of the fibronectin type-III domain-containing (FNDC) protein family in human cancers: A Review
Source: Medicine (Baltimore). 2022 Dec 9;101(49):e31854. doi: 10.1097/MD.0000000000031854 (PMC9750624; doi:10.1097/MD.0000000000031854)
Supplement: Supplementary file 3 [file medi-101-e31854-s003.pdf]

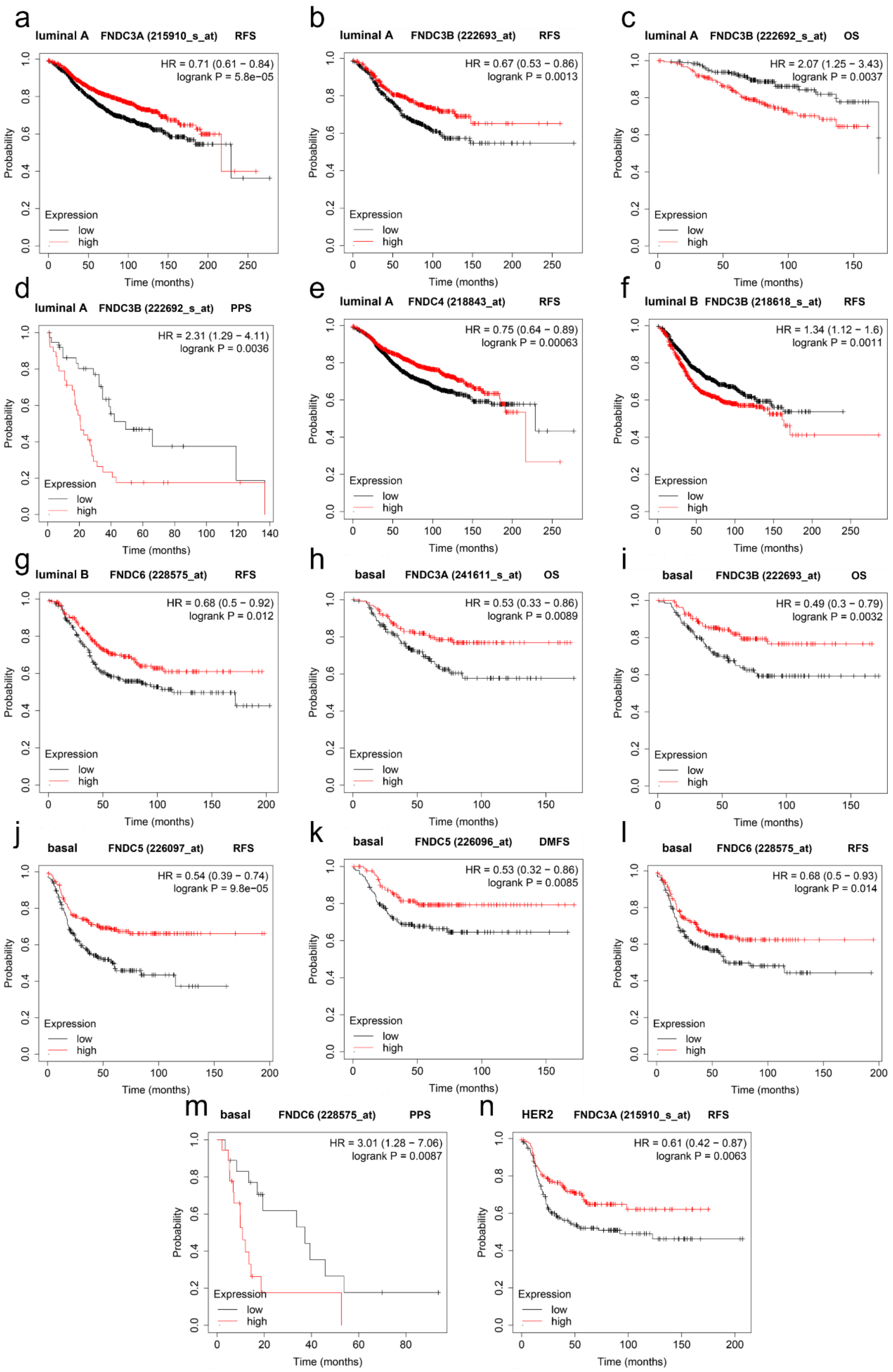


**Supplementary Fig 3.** Survival curves of the FNDC family in different St Gallen subtypes of breast cancer. Survival curves of the FNDC family in luminal A breast

cancer (a-e). Survival curves of the FNDC family in luminal B breast cancer (f-g). Survival curves of the FNDC family in basal breast cancer (h-m). Survival curves of FNDC3A (j) in HER2 breast cancer (n).
