# Supplementary material for: Expression and prognosis analyses of the fibronectin type-III domain-containing (FNDC) protein family in human cancers: A Review
Source: Medicine (Baltimore). 2022 Dec 9;101(49):e31854. doi: 10.1097/MD.0000000000031854 (PMC9750624; doi:10.1097/MD.0000000000031854)
Supplement: Supplementary file 4 [file medi-101-e31854-s004.pdf]

Table A. Survival analyses of FNDC family in breast cancer.

Gene	Dataset	Affymetrix ID/ Probe ID	Survival outcome	HR	95% CI	p-value
**Kaplan-Meier**						
**FNDC1**		226930_at	RFS	1.01	0.87 - 1.17	0.9328
			OS	1.13	0.87 - 1.48	0.3588
			DMFS	1.57	1.2 - 2.05	0.0009
			PPS	0.94	0.66 - 1.34	0.7426
**FNDC3A**		202304_at	RFS	0.81	0.73 - 0.9	4.2e-5
			OS	0.73	0.6 - 0.89	0.0013
			DMFS	0.71	0.61 - 0.83	1.4e-5
			PPS	1.12	0.89 - 1.42	0.33
		215910_s_at	RFS	0.76	0.69 - 0.84	1.1e-7
			OS	0.77	0.64 - 0.93	0.0062
			DMFS	0.88	0.75 - 1.02	0.093
			PPS	0.99	0.79 - 1.25	0.9311
		241611_s_at	RFS	0.89	0.77 - 1.04	0.1362
			OS	0.8	0.61 - 1.04	0.0945
			DMFS	0.97	0.75 - 1.27	0.8507
			PPS	0.85	0.6 - 1.22	0.378
**FNDC3B**		218618_s_at	RFS	1.3	1.17 - 1.44	4.3e-7
			OS	0.97	0.8 - 1.17	0.7272
			DMFS	1.08	0.92 - 1.26	0.3474
			PPS	1.29	1.02 - 1.63	0.0305
		222692_s_at	RFS	1.27	1.09 - 1.48	0.0019
			OS	1.39	1.06 - 1.82	0.0154
			DMFS	1.18	0.91 - 1.54	0.2086
			PPS	1.43	1 - 2.03	0.0462
		222693_at	RFS	0.86	0.74 - 0.99	0.0424
			OS	0.96	0.73 - 1.25	0.7546
			DMFS	1.21	0.93 - 1.58	0.1512
			PPS	1.15	0.81 - 1.63	0.4465
		225032_at	RFS	1.03	0.89 - 1.2	0.6785
			OS	1.24	0.95 - 1.62	0.1169
			DMFS	1.39	1.07 - 1.82	0.0139
			PPS	1.03	0.73 - 1.47	0.85
**FNDC4**		218843_at	RFS	0.91	0.82 - 1.01	0.0687
			OS	0.94	0.78 - 1.14	0.5376
			DMFS	0.99	0.85 - 1.16	0.9278
			PPS	0.93	0.74 - 1.17	0.5208
**FNDC5**		226096_at	RFS	0.86	0.74 - 1	0.0525
			OS	0.7	0.54 - 0.92	0.0112
			DMFS	0.79	0.61 - 1.04	0.0889

			PPS	0.81	0.57 - 1.16	0.2525
		226097_at	RFS	0.69	0.59 - 0.8	1.2e-6
			OS	1.02	0.78 - 1.33	0.901
			DMFS	0.8	0.61 - 1.04	0.0912
			PPS	1.17	0.82 - 1.66	0.3791
**FNDC6**		228575_at	RFS	0.8	0.69 - 0.94	0.0047
			OS	1.16	0.89 - 1.52	0.2675
			DMFS	1.39	1.07 - 1.82	0.0141
			PPS	1.42	1 - 2.02	0.0511
**FNDC7**		240837_at	RFS	0.97	0.84 - 1.13	0.7303
			OS	1.13	0.86 - 1.48	0.3739
			DMFS	1.01	0.78 - 1.32	0.934
			PPS	0.98	0.69 - 1.39	0.9078
**FNDC8**		220499_at	RFS	0.94	0.85 - 1.04	0.2578
			OS	0.97	0.81 - 1.17	0.7761
			DMFS	1.04	0.89 - 1.22	0.5847
			PPS	0.91	0.72 - 1.15	0.4279
**PrognoScan**						
**FNDC4**	GSE19615	218843_at	Distant Metastasis Free Survival	0.75	0.23 - 2.47	0.632147
	GSE12276	218843_at	Relapse Free Survival	0.92	0.80 - 1.06	0.263502
	GSE6532-GPL570	218843_at	Relapse Free Survival	0.54	0.17 - 1.72	0.297777
	GSE6532-GPL570	218843_at	Distant Metastasis Free Survival	0.54	0.17 - 1.72	0.297777
	GSE9195	218843_at	Relapse Free Survival	0.83	0.14 - 4.75	0.832954
	GSE9195	218843_at	Distant Metastasis Free Survival	0.35	0.05 - 2.48	0.292837
	GSE12093	218843_at	Distant Metastasis Free Survival	0.79	0.38 - 1.65	0.534922
	GSE11121	218843_at	Distant Metastasis Free Survival	0.78	0.47 - 1.31	0.356354
	GSE1378	12863	Relapse Free Survival	1.14	0.80 - 1.63	0.455452
	GSE1379	12863	Relapse Free Survival	1.13	0.69 - 1.83	0.62741
	GSE2034	218843_at	Distant Metastasis Free Survival	1.05	0.76 - 1.47	0.756521
	GSE1456-GPL96	218843_at	Overall Survival	0.37	0.16 - 0.85	0.018763
	GSE1456-GPL96	218843_at	Relapse Free Survival	0.32	0.13 - 0.75	0.008664
	GSE1456-GPL96	218843_at	Disease Specific Survival	0.28	0.10 - 0.77	0.013953
	GSE7378	218843_at	Disease Free Survival	0.55	0.11 - 2.73	0.465838
	E-TABM-158	218843_at	Distant Metastasis Free Survival	0.84	0.31 - 2.28	0.736707
	E-TABM-158	218843_at	Overall Survival	0.65	0.28 - 1.51	0.316457
	E-TABM-158	218843_at	Relapse Free Survival	0.65	0.28 - 1.51	0.316457
	E-TABM-158	218843_at	Disease Specific Survival	0.68	0.25 - 1.82	0.440769
	GSE3494-GPL96	218843_at	Disease Specific Survival	0.57	0.27 - 1.19	0.13478
	GSE4922-GPL96	218843_at	Disease Free Survival	0.85	0.49 - 1.49	0.575321
	GSE2990	218843_at	Distant Metastasis Free Survival	0.81	0.12 - 5.31	0.826306
	GSE2990	218843_at	Relapse Free Survival	0.80	0.19 - 3.36	0.765164
	GSE2990	218843_at	Distant Metastasis Free Survival	0.91	0.56 - 1.49	0.717229
	GSE2990	218843_at	Relapse Free Survival	0.92	0.62 - 1.38	0.699429
	GSE7390	218843_at	Relapse Free Survival	0.85	0.69 - 1.06	0.160241

GSE7390	218843_at	Distant Metastasis Free Survival	0.92	0.71 - 1.19	0.521864
GSE7390	218843_at	Overall Survival	0.91	0.69 - 1.19	0.481556
GSE19615	240837_at	Distant Metastasis Free Survival	1.39	0.40 - 4.83	0.599709
GSE12276	240837_at	Relapse Free Survival	0.95	0.83 - 1.09	0.468378
GSE6532-GPL570	240837_at	Distant Metastasis Free Survival	1.08	0.20 - 5.77	0.92576
GSE6532-GPL570	240837_at	Relapse Free Survival	1.08	0.20 - 5.77	0.92576
GSE9195	240837_at	Relapse Free Survival	0.58	0.05 - 6.85	0.667877
GSE9195	240837_at	Distant Metastasis Free Survival	0.12	0.01 - 1.96	0.136124
GSE1456-GPL97	240837_at	Overall Survival	0.72	0.45 - 1.15	0.168345
GSE1456-GPL97	240837_at	Relapse Free Survival	0.95	0.58 - 1.54	0.822416
GSE1456-GPL97	240837_at	Disease Specific Survival	0.69	0.40 - 1.21	0.195238
GSE3494-GPL97	240837_at	Disease Specific Survival	2.33	1.39 - 3.92	0.001327
GSE4922-GPL97	240837_at	Disease Free Survival	1.66	1.08 - 2.54	0.019985
GSE19615	220499_at	Distant Metastasis Free Survival	33.7	3.89 - 293.44	0.001419
GSE12276	220499_at	Relapse Free Survival	0.94	0.80 - 1.11	0.482091
GSE6532-GPL570	220499_at	Relapse Free Survival	3.49	0.79 - 15.47	0.099262
GSE6532-GPL570	220499_at	Distant Metastasis Free Survival	3.49	0.79 - 15.47	0.099262
GSE9195	220499_at	Distant Metastasis Free Survival	0.43	0.02 - 7.75	0.567187
GSE9195	220499_at	Relapse Free Survival	2.00	0.15 - 25.89	0.595733
GSE12093	220499_at	Distant Metastasis Free Survival	0.88	0.49 - 1.56	0.650311
GSE11121	220499_at	Distant Metastasis Free Survival	1.67	1.16 - 2.42	0.006296
GSE1378	3785	Relapse Free Survival	1.50	0.75 - 3.00	0.252075
GSE1379	3785	Relapse Free Survival	1.25	0.91 - 1.71	0.16162
GSE2034	220499_at	Distant Metastasis Free Survival	0.99	0.73 - 1.34	0.946469
GSE1456-GPL96	220499_at	Overall Survival	1.32	0.78 - 2.21	0.301935
GSE1456-GPL96	220499_at	Disease Specific Survival	1.60	0.87 - 2.92	0.127788
GSE1456-GPL96	220499_at	Relapse Free Survival	1.74	1.04 - 2.91	0.034959
GSE7378	220499_at	Disease Free Survival	0.25	0.01 - 8.56	0.440857
E-TABM-158	220499_at	Distant Metastasis Free Survival	3.22	0.48 - 21.54	0.227206
E-TABM-158	220499_at	Overall Survival	2.28	0.45 - 11.53	0.320215
E-TABM-158	220499_at	Relapse Free Survival	2.28	0.45 - 11.53	0.320215
E-TABM-158	220499_at	Disease Specific Survival	2.57	0.38 - 17.37	0.333272
GSE3494-GPL96	220499_at	Disease Specific Survival	0.99	0.62 - 1.57	0.960212
GSE4922-GPL96	220499_at	Disease Free Survival	1.14	0.80 - 1.63	0.476906
GSE2990	220499_at	Distant Metastasis Free Survival	0.56	0.17 - 1.83	0.341506
GSE2990	220499_at	Relapse Free Survival	0.83	0.33 - 2.04	0.678635
GSE2990	220499_at	Distant Metastasis Free Survival	1.04	0.61 - 1.77	0.890866
GSE2990	220499_at	Relapse Free Survival	0.98	0.63 - 1.52	0.931869
GSE7390	220499_at	Relapse Free Survival	1.01	0.84 - 1.22	0.885175
GSE7390	220499_at	Distant Metastasis Free Survival	0.99	0.79 - 1.24	0.93668
GSE7390	220499_at	Overall Survival	0.92	0.72 - 1.18	0.500173

HR, hazard ratio; CI, confidence interval; OS, overall survival; RFS, relapse free survival; DMFS, distant metastasis free survival; PPS, post progression survival. All of the data were obtained from the Kaplan-Meier Plotter and PrognoScan databases. The data with statistical significance were marked in red.
