# Supplementary material for: Expression and prognosis analyses of the fibronectin type-III domain-containing (FNDC) protein family in human cancers: A Review
Source: Medicine (Baltimore). 2022 Dec 9;101(49):e31854. doi: 10.1097/MD.0000000000031854 (PMC9750624; doi:10.1097/MD.0000000000031854)
Supplement: Supplementary file 5 [file medi-101-e31854-s005.pdf]

Table B. Survival analyses of FNDC family in lung cancer.

Table B1. Survival analyses of FNDC family in lung cancer from the Kaplan-Meier Plotter database.

Gene	Affymetrix ID	Survival outcome	Lung Adenocarcinoma			Squamous Cell Lung Carcinoma		
			HR	95% CI	p-value	HR	95% CI	p-value
**FNDC1**	226930_at	FP	1.25	0.91 - 1.73	0.1707	0.7	0.42 - 1.17	0.1693
		OS	1.25	0.98 - 1.6	0.0666	0.8	0.59 - 1.09	0.1585
		PPS	0.73	0.45 - 1.2	0.2136	1.2	0.43 - 3.35	0.7297
**FNDC3A**	202304_at	FP	0.59	0.43 - 0.81	0.001	1.31	0.78 - 2.19	0.3018
		OS	0.46	0.36 - 0.58	7.3e-11	0.99	0.78 - 1.26	0.9533
		PPS	0.6	0.38 - 0.96	0.0331	0.97	0.35 - 2.7	0.9583
	215910_s_at	FP	1.2	0.88 - 1.63	0.2607	1.81	1.07 - 3.06	0.0239
		OS	1.31	1.04 - 1.67	0.0243	0.94	0.74 - 1.19	0.5815
		PPS	0.86	0.54 - 1.38	0.5345	4.1	1.05 - 16.07	0.0297
	241611_s_at	FP	0.96	0.69 - 1.32	0.7954	0.52	0.31 - 0.88	0.0128
		OS	1.39	1.09 - 1.77	0.0077	0.8	0.59 - 1.09	0.1579
		PPS	1.07	0.66 - 1.75	0.7838	1.51	0.5 - 4.54	0.4622
**FNDC3B**	218618_s_at	FP	1.56	1.14 - 2.13	0.0056	1.35	0.8 - 2.26	0.256
		OS	1.29	1.02 - 1.63	0.0339	0.91	0.72 - 1.16	0.447
		PPS	0.85	0.53 - 1.35	0.484	2.13	0.69 - 6.58	0.1799
	222692_s_at	FP	1.7	1.22 - 2.35	0.0014	1	0.6 - 1.66	0.9901
		OS	1.2	0.94 - 1.53	0.1381	1.15	0.84 - 1.56	0.3846
		PPS	0.83	0.51 - 1.35	0.4453	1.18	0.43 - 3.29	0.7462
	222693_at	FP	1.22	0.88 - 1.68	0.2256	0.8	0.48 - 1.34	0.3952
		OS	1.35	1.06 - 1.72	0.0141	0.95	0.7 - 1.29	0.7455
		PPS	0.98	0.6 - 1.6	0.9499	1.28	0.44 - 3.74	0.6494
	225032_at	FP	0.72	0.52 - 1	0.0491	1.21	0.72 - 2.02	0.467
		OS	0.61	0.48 - 0.79	8.4e-5	1.03	0.75 - 1.42	0.8459
		PPS	0.68	0.42 - 1.12	0.1261	0.69	0.24 - 1.96	0.4872
**FNDC4**	218843_at	FP	1.78	1.29 - 2.45	0.0003	1.32	0.79 - 2.21	0.2934
		OS	1.42	1.12 - 1.79	0.0034	0.87	0.69 - 1.11	0.2661
		PPS	1.31	0.82 - 2.11	0.2588	0.45	0.16 - 1.28	0.1265
**FNDC5**	226096_at	FP	1.19	0.86 - 1.65	0.2804	1.08	0.65 - 1.81	0.7568
		OS	0.67	0.52 - 0.85	0.001	0.76	0.56 - 1.04	0.0825
		PPS	0.72	0.44 - 1.17	0.1842	2.27	0.75 - 6.81	0.1346
	226097_at	FP	1.65	1.19 - 2.29	0.0024	1.3	0.77 - 2.17	0.3232
		OS	1.36	1.07 - 1.73	0.0124	0.78	0.57 - 1.07	0.1292
		PPS	1.73	1.06 - 2.83	0.0278	1.22	0.44 - 3.39	0.7079
**FNDC6**	228575_at	FP	1.97	1.41 - 2.74	4.3e-5	1.31	0.78 - 2.19	0.3037
		OS	2.07	1.61 - 2.65	4.6e-9	0.95	0.7 - 1.29	0.7417
		PPS	0.96	0.59 - 1.56	0.8613	0.49	0.17 - 1.4	0.1749

HR, hazard ratio; CI, confidence interval; FP, first progression; OS, overall survival; PPS, post progression survival. All of the data were obtained from the Kaplan-Meier Plotter database. The data with statistical significance were marked in red.

Table B2. Survival analyses of FNDC1 in lung cancer from the PrognoScan database.

Gene	Dataset	Subtype	Probe ID	Survival outcome	HR	95% CI	p-value
**FNDC1**	GSE31210	Adenocarcinoma	226930_at	Overall Survival	1.62	1.18 - 2.23	0.002882
	GSE31210	Adenocarcinoma	226930_at	Relapse Free Survival	1.66	1.31 - 2.11	2.96E-05
	GSE3141	NSCLC	226930_at	Overall Survival	1.19	0.91 - 1.57	0.202156
	GSE8894	NSCLC	226930_at	Relapse Free Survival	1.07	0.93 - 1.21	0.345178
	GSE17710	Squamous cell carcinoma	20808	Relapse Free Survival	0.95	0.78 - 1.15	0.577701
	GSE17710	Squamous cell carcinoma	20808	Overall Survival	0.93	0.77 - 1.14	0.491924

HR, hazard ratio; CI, confidence interval. All of the data were obtained from the PrognoScan database. The data with statistical significance were marked in red.
