# Supplementary material for: Expression and prognosis analyses of the fibronectin type-III domain-containing (FNDC) protein family in human cancers: A Review
Source: Medicine (Baltimore). 2022 Dec 9;101(49):e31854. doi: 10.1097/MD.0000000000031854 (PMC9750624; doi:10.1097/MD.0000000000031854)
Supplement: Supplementary file 6 [file medi-101-e31854-s006.pdf]

Table C. Survival analyses of FNDC family in gastric cancer.

Gene	Affymetrix ID	Survival outcome	HR	95% CI	p-value
**FNDC1**	226930_at	OS	1.78	1.42 - 2.23	3.8e-7
		FP	1.87	1.46 - 2.4	5.7e-7
		PPS	2.1	1.58 - 2.79	2.0e-7
**FNDC3A**	202304_at	OS	0.58	0.49 - 0.69	3.9e-10
		FP	0.52	0.42 - 0.63	9.5e-11
		PPS	0.41	0.32 – 0.52	2.3e-14
	215910_s_at	OS	1.51	1.25 - 1.82	1.9e-5
		FP	1.42	1.16 - 1.75	0.0008
		PPS	2.09	1.68 - 2.62	3.0e-11
	241611_s_at	OS	0.76	0.6 - 0.96	0.0199
		FP	0.83	0.64 - 1.09	0.178
		PPS	0.77	0.58 - 1.03	0.0813
**FNDC3B**	218618_s_at	OS	1.59	1.35 - 1.89	5.5e-8
		FP	1.73	1.41 - 2.11	6.7e-8
		PPS	1.97	1.56 - 2.48	6.1e-9
	222692_s_at	OS	0.67	0.54 - 0.83	0.0002
		FP	0.7	0.55 - 0.89	0.0035
		PPS	0.64	0.49 - 0.84	0.0011
	222693_at	OS	0.66	0.51 - 0.86	0.0019
		FP	0.72	0.56 - 0.92	0.0097
		PPS	0.66	0.48 - 0.9	0.0082
	225032_at	OS	0.85	0.69 - 1.06	0.1542
		FP	1.13	0.86 - 1.49	0.3791
		PPS	0.79	0.58 - 1.08	0.1345
**FNDC4**	218843_at	OS	1.9	1.55 - 2.34	4.5e-10
		FP	1.81	1.45 - 2.27	1.6e-7
		PPS	2.27	1.78 - 2.89	8.0e-12
**FNDC5**	226096_at	OS	1.82	1.45 - 2.29	2.0e-7
		FP	1.74	1.36 - 2.23	1.1e-5
		PPS	2.1	1.6 - 2.76	5.2e-8
	226097_at	OS	1.76	1.41 - 2.2	5.2e-7
		FP	1.44	1.13 - 1.83	0.0027
		PPS	2.32	1.75 - 3.07	1.8e-9
**FNDC6**	228575_at	OS	1.43	1.13 - 1.8	0.0025
		FP	1.3	0.98 - 1.71	0.0645
		PPS	1.4	1.02 - 1.92	0.0367
**FNDC7**	240837_at	OS	1.47	1.18 - 1.83	0.0006
		FP	1.52	1.19 - 1.93	0.0007
		PPS	2.27	1.7 - 3.02	9.9e-9
**FNDC8**	220499_at	OS	1.35	1.1 - 1.66	0.0033
		FP	1.3	1.06 - 1.6	0.0124
		PPS	1.34	1.07 - 1.69	0.0119

HR, hazard ratio; CI, confidence interval; FP, first progression; OS, overall survival; PPS, post progression survival. All of the data were obtained from the Kaplan-Meier Plotter database. The data with statistical significance were marked in red.
