# Supplementary material for: Expression and prognosis analyses of the fibronectin type-III domain-containing (FNDC) protein family in human cancers: A Review
Source: Medicine (Baltimore). 2022 Dec 9;101(49):e31854. doi: 10.1097/MD.0000000000031854 (PMC9750624; doi:10.1097/MD.0000000000031854)
Supplement: Supplementary file 7 [file medi-101-e31854-s007.pdf]

Table D. Survival analyses of FNDC family in ovarian cancer.

Gene	Dataset	Affymetrix ID/ Probe ID	Survival outcome	HR	95% CI	p-value
**Kaplan-Meier**						
**FNDC1**		226930_at	PFS	1.57	1.29 - 1.92	6.7e-6
			OS	1.39	1.13 - 1.72	0.0022
			PPS	1.24	0.96 - 1.6	0.103
**FNDC3A**		202304_at	PFS	1.23	1.07 - 1.42	0.0035
			OS	1.24	1.07 - 1.43	0.0043
			PPS	1.19	1 – 1.41	0.0495
		215910_s_at	PFS	1.08	0.95 - 1.23	0.2446
			OS	1.08	0.93 - 1.25	0.3047
			PPS	1.22	1.02 - 1.45	0.0269
		241611_s_at	PFS	0.84	0.69 - 1.02	0.0808
			OS	0.92	0.75 - 1.12	0.397
			PPS	1.33	1.02 - 1.74	0.0372
**FNDC3B**		218618_s_at	PFS	1.09	0.95 - 1.24	0.2248
			OS	0.9	0.79 - 1.02	0.1006
			PPS	1.14	0.96 - 1.37	0.1447
		222692_s_at	PFS	1.2	0.97 - 1.49	0.0959
			OS	1.34	1.08 - 1.65	0.0065
			PPS	0.78	0.6 - 1.02	0.0691
		222693_at	PFS	1.19	0.96 - 1.48	0.1154
			OS	1.62	1.27 - 2.07	7.7e-5
			PPS	1.26	0.96 - 1.66	0.0989
		225032_at	PFS	0.88	0.73 - 1.06	0.1845
			OS	1.34	1.08 - 1.65	0.0071
			PPS	1.35	1.04 - 1.75	0.0254
**FNDC4**		218843_at	PFS	0.91	0.8 - 1.04	0.1529
			OS	0.92	0.81 - 1.05	0.2118
			PPS	1.22	1.03 - 1.44	0.0213
**FNDC5**		226096_at	PFS	1.31	1.05 - 1.63	0.0169
			OS	1.34	1.07 - 1.68	0.0098
			PPS	1.37	1.06 - 1.77	0.0152
		226097_at	PFS	1.42	1.16 - 1.75	0.0008
			OS	1.24	1 - 1.54	0.0497
			PPS	1.12	0.88 - 1.41	0.3615
**FNDC6**		228575_at	PFS	1.26	1.04 - 1.52	0.0156
			OS	1.33	1.09 - 1.63	0.0056
			PPS	0.75	0.58 - 0.98	0.0325
**FNDC7**		240837_at	PFS	1.32	1.09 - 1.6	0.0049
			OS	1.39	1.13 - 1.71	0.0018
			PPS	1.21	0.95 - 1.53	0.1166
**FNDC8**		220499_at	PFS	0.78	0.69 - 0.89	0.0002

			OS	0.85	0.74 - 0.96	0.0116
			PPS	0.9	0.76 - 1.07	0.2346
**PrognoScan**						
**FNDC4**	GSE9891	218843_at	Overall Survival	1.15	0.70 - 1.90	0.575621
	DUKE-OC	218843_at	Overall Survival	6.61	1.62 - 27.02	0.008563
	GSE8841	12614	Overall Survival	1.19	0.63 - 2.27	0.59497
	GSE26712	218843_at	Disease Free Survival	1.11	0.75 - 1.64	0.618656
	GSE26712	218843_at	Overall Survival	1.20	0.78 - 1.83	0.410771
	GSE17260	A_23_P16834	Overall Survival	0.86	0.65 - 1.13	0.283563
	GSE17260	A_23_P16834	Progression Free Survival	1.00	0.81 - 1.23	0.971289
	GSE14764	218843_at	Overall Survival	0.77	0.48 - 1.25	0.297653

HR, hazard ratio; CI, confidence interval; PFS, progression free survival; OS, overall survival; PPS, post progression survival. All of the data were obtained from the Kaplan-Meier Plotter and PrognoScan databases. The data with statistical significance were marked in red.

**FNDC3B**	1559975_at	RFS	1.15 (0.92 - 1.44)	0.2113	1.16 (0.99 - 1.37)	0.0721	1.34 (1.12 - 1.6)	0.0011	1.08 (0.76 - 1.54)	0.6614
		OS	0.8 (0.55 - 1.18)	0.2593	0.87 (0.63 - 1.19)	0.3755	1.21 (0.86 - 1.72)	0.2735	0.96 (0.55 - 1.7)	0.9014
		DMFS	0.69 (0.51 - 0.95)	0.0221	1.16 (0.89 - 1.5)	0.269	1.05 (0.79 - 1.39)	0.7489	1.47 (0.89 - 2.42)	0.1289
		PPS	0.86 (0.49 - 1.51)	0.5992	1.26 (0.88 - 1.81)	0.2046	1.45 (0.95 - 2.23)	0.0839	1.18 (0.56 - 2.48)	0.6649
	222692_s_at	RFS	0.81 (0.59 - 1.1)	0.167	1.11 (0.87 - 1.42)	0.3847	1.15 (0.85 - 1.55)	0.374	1.01 (0.65 - 1.56)	0.9596
		OS	0.88 (0.56 - 1.4)	0.5959	2.07 (1.25 - 3.43)	0.0037	0.85 (0.46 - 1.58)	0.6124	0.74 (0.37 - 1.47)	0.3819
		DMFS	0.69 (0.43 - 1.11)	0.1271	1.03 (0.62 - 1.7)	0.9096	0.81 (0.46 - 1.46)	0.4874	1.02 (0.56 - 1.85)	0.9523
		PPS	0.88 (0.4 – 1.94)	0.7523	2.31 (1.29 - 4.11)	0.0036	1.44 (0.69 - 3)	0.3307	0.91 (0.39 - 2.11)	0.8322
	222693_at	RFS	0.73 (0.54 - 1)	0.0478	0.67 (0.53 - 0.86)	0.0013	0.69 (0.51 - 0.94)	0.0175	0.59 (0.38 - 0.91)	0.0168
		OS	0.49 (0.3 - 0.79)	0.0032	1.01 (0.63 - 1.62)	0.9722	0.8 (0.43 - 1.47)	0.4685	0.95 (0.48 - 1.87)	0.8737
		DMFS	0.71 (0.45 - 1.14)	0.1583	1.67 (0.99 - 2.8)	0.05	1.03 (0.58 - 1.84)	0.9252	0.8 (0.44 - 1.46)	0.4618
		PPS	0.87 (0.39 - 1.95)	0.7377	1.2 (0.68 - 2.14)	0.5251	1.15 (0.55 - 2.38)	0.7101	1.59 (0.68 - 3.69)	0.2788
	225032_at	RFS	1.07 (0.79 - 1.46)	0.6531	0.79 (0.62 - 1.01)	0.0576	0.89 (0.66 - 1.21)	0.4625	0.94 (0.61 - 1.45)	0.7783
		OS	0.85 (0.54 - 1.35)	0.4943	1.43 (0.89 - 2.31)	0.1385	0.83 (0.45 - 1.53)	0.55	1.08 (0.54 - 2.14)	0.8295
		DMFS	1.44 (0.9 - 2.3)	0.1302	1.72 (1.03 - 2.88)	0.0357	0.85 (0.48 - 1.53)	0.5963	1.19 (0.65 - 2.17)	0.5736
		PPS	0.88 (0.4 - 1.93)	0.747	1.08 (0.61 - 1.91)	0.7833	1.33 (0.64 - 2.77)	0.4398	0.75 (0.32 - 1.74)	0.5001
**FNDC4**	218843_at	RFS	1.2 (0.96 - 1.5)	0.1054	0.75 (0.64 - 0.89)	0.0006	0.93 (0.78 - 1.11)	0.4069	1.06 (0.75 - 1.52)	0.7309
		OS	1.6 (1.09 - 2.36)	0.0157	0.74 (0.54 - 1.02)	0.063	0.93 (0.65 - 1.31)	0.6705	1.16 (0.66 - 2.05)	0.6069
		DMFS	1.15 (0.84 - 1.57)	0.3848	0.79 (0.61 - 1.03)	0.0828	1.25 (0.94 - 1.65)	0.1224	1.13 (0.69 - 1.85)	0.6335
		PPS	1.36 (0.78 - 2.36)	0.2768	0.86 (0.6 - 1.23)	0.4116	0.9 (0.59 - 1.39)	0.637	0.67 (0.31 - 1.42)	0.2937
**FNDC5**	226096_at	RFS	0.88 (0.65 - 1.19)	0.4038	0.86 (0.68 - 1.1)	0.2366	0.92 (0.68 - 1.24)	0.5682	1.08 (0.69 - 1.66)	0.7437
		OS	0.85 (0.54 - 1.36)	0.5038	0.78 (0.48 - 1.25)	0.2991	0.56 (0.3 - 1.06)	0.0719	1.3 (0.65 - 2.57)	0.4545
		DMFS	0.53 (0.32 - 0.86)	0.0085	0.92 (0.56 - 1.53)	0.7614	1.13 (0.63 - 2.02)	0.6796	1.41 (0.77 - 2.57)	0.2663
		PPS	0.85 (0.38 - 1.87)	0.6765	0.96 (0.55 - 1.67)	0.8856	0.56 (0.27 - 1.18)	0.1234	2.06 (0.87 - 4.87)	0.0933
	226097_at	RFS	0.54 (0.39 - 0.74)	9.8e-5	0.82 (0.64 - 1.04)	0.1051	0.74 (0.54 - 1)	0.0471	0.98 (0.64 - 1.52)	0.9456
		OS	1.2 (0.75 - 1.9)	0.4467	1.17 (0.73 - 1.89)	0.5203	1.02 (0.55 - 1.89)	0.9488	1.15 (0.58 - 2.29)	0.6852
		DMFS	0.7 (0.44 - 1.12)	0.1318	0.91 (0.55 - 1.51)	0.7247	1.01 (0.57 - 1.8)	0.9712	1.25 (0.68 - 2.29)	0.4689
		PPS	1.41 (0.64 - 3.11)	0.3976	1.1 (0.63 - 1.92)	0.7366	1.29 (0.62 - 2.69)	0.4975	1.34 (0.57 - 3.13)	0.4971
**FNDC6**	228575_at	RFS	0.68 (0.5 - 0.93)	0.0139	0.91 (0.72 - 1.16)	0.4468	0.68 (0.5 - 0.92)	0.0123	1.1 (0.71 - 1.71)	0.6628
		OS	1.04 (0.66 - 1.66)	0.8565	1.02 (0.64 - 1.64)	0.9248	1.11 (0.6 - 2.06)	0.7328	2.23 (1.08 - 4.61)	0.026

		DMFS	0.99 (0.62 - 1.57)	0.9607	1.58 (0.94 - 2.64)	0.0796	1.34 (0.75 - 2.41)	0.3189	1.64 (0.89 - 3.03)	0.1107
		PPS	3.01 (1.28 - 7.06)	0.0087	1.18 (0.67 - 2.08)	0.5644	1.03 (0.49 - 2.14)	0.9445	1.69 (0.73 - 3.92)	0.2192
**FNDC7**	240837_at	RFS	0.81 (0.59 - 1.1)	0.1691	0.94 (0.74 - 1.19)	0.5949	0.92 (0.68 - 1.25)	0.6099	1.22 (0.79 - 1.89)	0.368
		OS	1.18 (0.74 - 1.87)	0.4859	1.12 (0.7 - 1.8)	0.6269	1.45 (0.78 - 2.7)	0.2345	1.19 (0.6 - 2.36)	0.6107
		DMFS	1.1 (0.69 - 1.74)	0.7003	1.05 (0.64 -1.74)	0.8412	1.51 (0.84 - 2.71)	0.164	1.09 (0.6 - 1.99)	0.7673
		PPS	0.78 (0.35 - 1.71)	0.5291	0.85 (0.49 - 1.49)	0.5693	1.59 (0.75 - 3.38)	0.2247	1.37 (0.59 - 3.19)	0.4672
**FNDC8**	220499_at	RFS	1.01 (0.81 - 1.26)	0.9152	0.92 (0.78 - 1.09)	0.3382	0.93 (0.78 - 1.11)	0.4302	0.88 (0.62 - 1.26)	0.4945
		OS	1.23 (0.84 - 1.8)	0.276	0.86 (0.62 - 1.18)	0.3452	1.09 (0.77 - 1.54)	0.6438	1.18 (0.67 - 2.08)	0.5702
		DMFS	1.34 (0.98 - 1.84)	0.0619	0.96 (0.74 - 1.25)	0.7698	1.26 (0.95 - 1.67)	0.1023	0.97 (0.59 - 1.6)	0.9151
		PPS	1.24 (0.71 - 2.16)	0.4442	0.84 (0.58 - 1.2)	0.3359	1.08 (0.71 - 1.65)	0.7132	1.03 (0.49 - 2.18)	0.9308

HR, hazard ratio; CI, confidence interval; OS, overall survival; RFS, relapse free survival; DMFS, distant metastasis free survival; PPS, post progression survival. All of the data were obtained from the Kaplan-Meier Plotter database. The data with statistical significance were marked in red.
