# Supplementary material for: Expression and prognosis analyses of the fibronectin type-III domain-containing (FNDC) protein family in human cancers: A Review
Source: Medicine (Baltimore). 2022 Dec 9;101(49):e31854. doi: 10.1097/MD.0000000000031854 (PMC9750624; doi:10.1097/MD.0000000000031854)
Supplement: Supplementary file 8 [file medi-101-e31854-s008.pdf]

Table E. Survival analyses of FNDC family in liver cancer.

Gene	RNA-Seq ID	Survival outcome	HR	95% CI	p-value
**FNDC1**	84624	OS	0.76	0.51 - 1.12	0.1679
		RFS	0.57	0.4 - 0.8	0.0013
		PFS	0.64	0.47 - 0.87	0.0041
		DSS	0.71	0.43 - 1.17	0.1738
**FNDC3A**	22862	OS	0.56	0.4 - 0.8	0.0011
		RFS	0.71	0.51 - 0.99	0.0414
		PFS	0.73	0.54 – 0.99	0.0419
		DSS	0.57	0.37 - 0.9	0.0133
**FNDC3B**	64778	OS	1.42	0.98 - 2.05	0.0638
		RFS	0.69	0.49 - 0.98	0.0379
		PFS	0.8	0.58 - 1.1	0.162
		DSS	1.4	0.88 - 2.24	0.1531
**FNDC4**	64838	OS	1.46	1.03 - 2.08	0.0334
		RFS	1.17	0.84 - 1.62	0.3521
		PFS	1.17	0.83 - 1.65	0.382
		DSS	1.66	1.06 - 2.61	0.0249
**FNDC5**	252995	OS	0.68	0.47 - 0.98	0.0377
		RFS	0.61	0.42 - 0.88	0.0083
		PFS	0.62	0.45 - 0.87	0.0044
		DSS	0.73	0.46 - 1.16	0.1815
**FNDC6**	53833	OS	1.61	1.12 - 2.33	0.0098
		RFS	1.23	0.88 - 1.71	0.2177
		PFS	1.26	0.94 - 1.69	0.1177
		DSS	1.63	1.05 - 2.55	0.0282
**FNDC7**	163479	OS	0.46	0.32 - 0.65	6.4e-6
		RFS	0.61	0.44 - 0.84	0.0026
		PFS	0.6	0.44 - 0.82	0.0013
		DSS	0.36	0.23 - 0.56	1.9e-6
**FNDC8**	54752	OS	1.4	0.98 - 2	0.0629
		RFS	1.43	1.02 - 1.98	0.0345
		PFS	1.54	1.14 - 2.09	0.0046
		DSS	1.66	1.01 - 2.74	0.0436

HR, hazard ratio; CI, confidence interval; OS, overall survival; RFS, relapse free survival; PFS, progression free survival; DSS, disease specific survival. All of the data were obtained from the Kaplan-Meier Plotter database. The data with statistical significance were marked in red.
