# Supplementary material for: Expression and prognosis analyses of the fibronectin type-III domain-containing (FNDC) protein family in human cancers: A Review
Source: Medicine (Baltimore). 2022 Dec 9;101(49):e31854. doi: 10.1097/MD.0000000000031854 (PMC9750624; doi:10.1097/MD.0000000000031854)
Supplement: Supplementary file 9 [file medi-101-e31854-s009.pdf]

Table F. Survival analyses of FNDC family in colorectal cancer.

Gene	Dataset	Probe ID	Survival outcome	HR	95% CI	p-value
**FNDC1**	GSE17536	226930_at	Disease Free Survival	1.56	1.19 - 2.05	0.0013
	GSE17536	226930_at	Overall Survival	1.27	1.05 - 1.55	0.014055
	GSE17536	226930_at	Disease Specific Survival	1.47	1.18 - 1.84	0.00076
	GSE14333	226930_at	Disease Free Survival	1.36	1.11 - 1.67	0.002849
	GSE17537	226930_at	Disease Free Survival	1.25	0.92 - 1.70	0.154435
	GSE17537	226930_at	Disease Specific Survival	1.11	0.76 - 1.61	0.588269
	GSE17537	226930_at	Overall Survival	1.08	0.79 - 1.47	0.638512
**FNDC3A**	GSE12945	215910_s_at	Overall Survival	1.48	0.15 - 15.02	0.741786
	GSE12945	202304_at	Disease Free Survival	1.05	0.23 - 4.88	0.946646
	GSE12945	202304_at	Overall Survival	1.22	0.50 - 2.97	0.659721
	GSE12945	215910_s_at	Disease Free Survival	0.47	0.01 - 15.07	0.671733
	GSE17536	215910_s_at	Disease Specific Survival	1.17	0.63 - 2.17	0.621164
	GSE17536	238961_s_at	Overall Survival	0.69	0.18 - 2.64	0.585903
	GSE17536	238961_s_at	Disease Specific Survival	0.54	0.12 - 2.54	0.438417
	GSE17536	241611_s_at	Overall Survival	0.51	0.19 - 1.36	0.177709
	GSE17536	241611_s_at	Disease Specific Survival	0.47	0.15 - 1.48	0.197455
	GSE17536	238961_s_at	Disease Free Survival	1.18	0.18 - 7.52	0.864066
	GSE17536	241611_s_at	Disease Free Survival	0.72	0.18 - 2.93	0.64544
	GSE17536	202304_at	Disease Specific Survival	1.73	0.81 - 3.69	0.154003
	GSE17536	215910_s_at	Overall Survival	1.05	0.61 - 1.82	0.847487
	GSE17536	202304_at	Overall Survival	1.62	0.84 - 3.12	0.15241
	GSE17536	215910_s_at	Disease Free Survival	0.93	0.41 - 2.07	0.85069
	GSE17536	202304_at	Disease Free Survival	1.43	0.57 - 3.61	0.447061
	GSE14333	238961_s_at	Disease Free Survival	0.87	0.65 - 1.17	0.357186
	GSE14333	241611_s_at	Disease Free Survival	1.20	0.91 - 1.58	0.198904
	GSE14333	215910_s_at	Disease Free Survival	1.22	0.69 - 2.13	0.494589
	GSE14333	202304_at	Disease Free Survival	0.86	0.41 - 1.80	0.681842
	GSE17537	215910_s_at	Overall Survival	0.81	0.32 - 2.06	0.661707
	GSE17537	202304_at	Overall Survival	1.23	0.43 - 3.49	0.701417
	GSE17537	238961_s_at	Disease Free Survival	0.43	0.02 - 8.26	0.578608
	GSE17537	241611_s_at	Disease Free Survival	0.78	0.06 - 10.19	0.852224
	GSE17537	215910_s_at	Disease Free Survival	0.51	0.18 - 1.43	0.198388
	GSE17537	215910_s_at	Disease Specific Survival	0.43	0.14 - 1.35	0.148867
	GSE17537	238961_s_at	Disease Specific Survival	1.09	0.02 - 61.42	0.966773
	GSE17537	241611_s_at	Disease Specific Survival	0.48	0.02 - 10.44	0.639487
	GSE17537	202304_at	Disease Free Survival	1.20	0.39 - 3.69	0.753799
	GSE17537	202304_at	Disease Specific Survival	1.44	0.41 - 5.09	0.569691
	GSE17537	238961_s_at	Overall Survival	1.64	0.10 - 27.55	0.729727
	GSE17537	241611_s_at	Overall Survival	0.26	0.02 - 2.90	0.272649
**FNDC3B**	GSE12945	218618_s_at	Disease Free Survival	1.21	0.36 - 4.11	0.754307
	GSE12945	218618_s_at	Overall Survival	1.59	0.81 - 3.14	0.180137
	GSE17536	222693_at	Disease Specific Survival	1.12	0.71 - 1.77	0.62119

GSE17536	225032_at	Overall Survival	1.24	0.64 - 2.43	0.523572
GSE17536	218618_s_at	Disease Specific Survival	1.46	0.82 - 2.59	0.202285
GSE17536	222692_s_at	Overall Survival	1.39	0.78 - 2.49	0.263656
GSE17536	222692_s_at	Disease Specific Survival	1.12	0.57 - 2.18	0.744223
GSE17536	222693_at	Overall Survival	1.30	0.87 - 1.93	0.198133
GSE17536	242029_at	Overall Survival	0.84	0.45 - 1.58	0.586018
GSE17536	225032_at	Disease Free Survival	2.82	1.09 - 7.28	0.032654
GSE17536	242029_at	Disease Specific Survival	0.93	0.45 - 1.90	0.843798
GSE17536	222692_s_at	Disease Free Survival	1.96	0.85 - 4.55	0.115123
GSE17536	225032_at	Disease Specific Survival	1.21	0.56 - 2.62	0.625587
GSE17536	1569490_at	Overall Survival	1.17	0.35 - 3.83	0.800687
GSE17536	229865_at	Overall Survival	0.88	0.52 - 1.48	0.62737
GSE17536	222693_at	Disease Free Survival	1.56	0.90 - 2.72	0.113615
GSE17536	1569490_at	Disease Specific Survival	1.28	0.33 - 4.97	0.720558
GSE17536	229865_at	Disease Specific Survival	0.67	0.35 - 1.26	0.212839
GSE17536	218618_s_at	Overall Survival	1.36	0.83 - 2.24	0.226395
GSE17536	1569490_at	Disease Free Survival	2.26	0.43 - 11.75	0.333374
GSE17536	229865_at	Disease Free Survival	0.92	0.46 - 1.84	0.824333
GSE17536	218618_s_at	Disease Free Survival	1.78	0.86 - 3.67	0.121551
GSE17536	242029_at	Disease Free Survival	1.08	0.46 - 2.52	0.854421
GSE14333	225032_at	Disease Free Survival	2.82	1.17 - 6.79	0.020739
GSE14333	222692_s_at	Disease Free Survival	2.01	0.95 - 4.25	0.067691
GSE14333	222693_at	Disease Free Survival	1.39	0.81 - 2.39	0.226464
GSE14333	242029_at	Disease Free Survival	0.98	0.80 - 1.20	0.847894
GSE14333	1569490_at	Disease Free Survival	1.39	0.81 - 2.39	0.226129
GSE14333	229865_at	Disease Free Survival	0.87	0.61 - 1.25	0.456747
GSE14333	218618_s_at	Disease Free Survival	1.30	0.72 - 2.34	0.378546
GSE17537	242029_at	Overall Survival	0.36	0.05 - 2.67	0.320265
GSE17537	1569490_at	Overall Survival	0.61	0.03 - 10.90	0.735151
GSE17537	229865_at	Overall Survival	2.18	0.85 - 5.58	0.106157
GSE17537	218618_s_at	Overall Survival	1.08	0.46 - 2.55	0.864064
GSE17537	218618_s_at	Disease Free Survival	0.69	0.28 - 1.72	0.424876
GSE17537	222692_s_at	Disease Free Survival	4.29	1.02 - 18.13	0.047617
GSE17537	222693_at	Disease Free Survival	1.47	0.64 - 3.39	0.361582
GSE17537	222693_at	Disease Specific Survival	1.10	0.42 - 2.88	0.848246
GSE17537	218618_s_at	Disease Specific Survival	0.64	0.22 - 1.89	0.42095
GSE17537	222692_s_at	Disease Specific Survival	3.29	0.61 - 17.73	0.165946
GSE17537	242029_at	Disease Free Survival	0.19	[0.02 - 1.85]	0.152942
GSE17537	225032_at	Disease Free Survival	2.37	0.76 - 7.34	0.135006
GSE17537	1569490_at	Disease Free Survival	0.11	0.00 - 2.33	0.15489
GSE17537	229865_at	Disease Free Survival	1.70	0.60 - 4.81	0.315817
GSE17537	242029_at	Disease Specific Survival	0.15	0.01 - 1.79	0.134175
GSE17537	225032_at	Disease Specific Survival	1.84	0.47 - 7.18	0.381382
GSE17537	1569490_at	Disease Specific Survival	0.25	0.01 - 8.95	0.450169

	GSE17537	229865_at	Disease Specific Survival	1.61	0.39 - 6.67	0.510442
	GSE17537	225032_at	Overall Survival	3.03	1.10 - 8.39	0.032736
	GSE17537	222692_s_at	Overall Survival	5.46	1.52 - 19.54	0.009097
	GSE17537	222693_at	Overall Survival	2.17	0.95 - 4.95	0.066402
**FNDC4**	GSE12945	218843_at	Disease Free Survival	10.53	0.36 - 306.45	0.170978
	GSE12945	218843_at	Overall Survival	1.98	0.21 - 18.62	0.549579
	GSE17536	218843_at	Disease Free Survival	1.56	0.43 - 5.61	0.496505
	GSE17536	218843_at	Disease Specific Survival	0.89	0.29 - 2.70	0.831578
	GSE17536	218843_at	Overall Survival	0.87	0.33 - 2.29	0.784997
	GSE14333	218843_at	Disease Free Survival	1.09	0.80 - 1.48	0.59348
	GSE17537	218843_at	Disease Specific Survival	0.55	0.01 - 21.18	0.74523
	GSE17537	218843_at	Overall Survival	0.49	0.03 - 9.39	0.639262
	GSE17537	218843_at	Disease Free Survival	0.70	0.03 - 14.85	0.817277
**FNDC5**	GSE17536	226097_at	Overall Survival	0.13	0.03 - 0.65	0.01301
	GSE17536	226097_at	Disease Specific Survival	0.19	0.03 - 1.17	0.073146
	GSE17536	230646_at	Overall Survival	0.22	0.06 - 0.80	0.02079
	GSE17536	226096_at	Overall Survival	1.00	0.33 - 3.02	0.999237
	GSE17536	230646_at	Disease Specific Survival	0.16	0.04 - 0.69	0.013745
	GSE17536	226096_at	Disease Specific Survival	1.26	0.36 - 4.42	0.723639
	GSE17536	226097_at	Disease Free Survival	0.34	0.03 - 3.31	0.350459
	GSE17536	230646_at	Disease Free Survival	0.69	0.10 - 4.63	0.705087
	GSE17536	226096_at	Disease Free Survival	0.82	0.17 - 4.04	0.804497
	GSE14333	226097_at	Disease Free Survival	1.00	0.61 - 1.65	0.9914
	GSE14333	230646_at	Disease Free Survival	0.88	0.44 - 1.77	0.722477
	GSE14333	226096_at	Disease Free Survival	0.90	0.55 - 1.46	0.663915
	GSE17537	226097_at	Disease Free Survival	0.56	0.02 - 14.58	0.726689
	GSE17537	230646_at	Disease Free Survival	0.05	0.00 - 0.87	0.039689
	GSE17537	226096_at	Disease Free Survival	2.14	0.07 - 64.82	0.662158
	GSE17537	226097_at	Disease Specific Survival	0.56	0.01 - 24.89	0.765718
	GSE17537	230646_at	Disease Specific Survival	0.04	0.00 - 1.31	0.070596
	GSE17537	226096_at	Disease Specific Survival	0.41	0.01 - 26.33	0.676842
	GSE17537	226097_at	Overall Survival	1.04	0.05 - 21.07	0.981195
	GSE17537	230646_at	Overall Survival	0.03	0.00 - 0.44	0.010657
	GSE17537	226096_at	Overall Survival	1.80	0.07 - 48.95	0.725876
**FNDC6**	GSE17536	228575_at	Overall Survival	1.30	0.66 - 2.55	0.450413
	GSE17536	228575_at	Disease Specific Survival	1.18	0.52 - 2.69	0.684481
	GSE17536	228575_at	Disease Free Survival	1.30	0.51 - 3.30	0.57735
	GSE14333	228575_at	Disease Free Survival	0.99	0.66 - 1.51	0.975156
	GSE17537	228575_at	Disease Free Survival	1.86	0.50 - 6.98	0.354586
	GSE17537	228575_at	Disease Specific Survival	0.31	0.02 - 4.91	0.402457
	GSE17537	228575_at	Overall Survival	1.08	0.30 - 3.86	0.909177
**FNDC7**	GSE17536	240837_at	Disease Specific Survival	0.11	0.02 - 0.51	0.004922
	GSE17536	240837_at	Overall Survival	0.14	0.04 - 0.53	0.003851
	GSE17536	240837_at	Disease Free Survival	0.30	0.05 - 1.88	0.196533

	GSE14333	240837_at	Disease Free Survival	1.24	0.96 - 1.61	0.093934
	GSE17537	240837_at	Disease Free Survival	0.02	0.00 - 0.24	0.002196
	GSE17537	240837_at	Disease Specific Survival	0.04	0.00 - 0.60	0.018995
	GSE17537	240837_at	Overall Survival	0.12	0.01 - 1.09	0.059942
**FNDC8**	GSE12945	220499_at	Disease Free Survival	2.77	0.13 - 57.77	0.5104
	GSE12945	220499_at	Overall Survival	1.17	0.16 - 8.47	0.878038
	GSE17536	220499_at	Overall Survival	0.31	0.10 - 0.94	0.038835
	GSE17536	220499_at	Disease Free Survival	0.27	0.06 - 1.35	0.111498
	GSE17536	220499_at	Disease Specific Survival	0.24	0.07 - 0.89	0.032596
	GSE14333	220499_at	Disease Free Survival	1.01	0.72 - 1.42	0.949409
	GSE17537	220499_at	Disease Free Survival	0.02	0.00 - 0.45	0.012746
	GSE17537	220499_at	Disease Specific Survival	0.07	0.00 - 1.44	0.084014
	GSE17537	220499_at	Overall Survival	0.08	0.01 - 0.80	0.031564

HR, hazard ratio; CI, confidence interval. All of the data were obtained from the PrognoScan database. The data with statistical significance were marked in red.
