# Supplementary material for: Expression and prognosis analyses of the fibronectin type-III domain-containing (FNDC) protein family in human cancers: A Review
Source: Medicine (Baltimore). 2022 Dec 9;101(49):e31854. doi: 10.1097/MD.0000000000031854 (PMC9750624; doi:10.1097/MD.0000000000031854)
Supplement: Supplementary file 10 [file medi-101-e31854-s010.pdf]

Table G. Survival analyses of FNDC family in bladder, blood, brain and skin cancers.

Gene	Dataset	Probe ID	Survival outcome	HR	95% CI	p-value
**Bladder Cancer**						
**FNDC1**	GSE13507	ILMN_1734653	Overall Survival	1.48	1.01 - 2.18	0.046968
	GSE13507	ILMN_1734653	Disease Specific Survival	2.07	1.35 - 3.16	0.000799
**FNDC3A**	GSE5287	202304_at	Overall Survival	0.60	0.25 - 1.41	0.242015
	GSE5287	215910_s_at	Overall Survival	0.51	0.13 - 2.05	0.341281
	GSE13507	ILMN_1705111	Overall Survival	0.78	0.60 - 1.01	0.060751
	GSE13507	ILMN_1705111	Disease Specific Survival	0.64	0.45 - 0.92	0.016253
**FNDC3B**	GSE5287	218618_s_at	Overall Survival	0.76	0.41 - 1.38	0.365503
	GSE13507	ILMN_1753554	Overall Survival	1.23	0.87 - 1.74	0.234039
	GSE13507	ILMN_1753554	Disease Specific Survival	1.19	0.70 - 2.00	0.523411
**FNDC4**	GSE5287	218843_at	Overall Survival	1.53	0.53 - 4.42	0.431857
	GSE13507	ILMN_1663032	Overall Survival	1.07	0.80 - 1.45	0.635778
	GSE13507	ILMN_1663032	Disease Specific Survival	1.07	0.70 - 1.64	0.742267
**FNDC5**	GSE13507	ILMN_1761084	Overall Survival	1.02	0.71 - 1.47	0.906942
	GSE13507	ILMN_1761084	Disease Specific Survival	1.68	1.12 - 2.52	0.012763
**FNDC6**	GSE13507	ILMN_1765668	Overall Survival	1.32	1.04 - 1.68	0.020857
	GSE13507	ILMN_1765668	Disease Specific Survival	1.50	1.11 - 2.03	0.008515
**FNDC7**	GSE13507	ILMN_1742179	Overall Survival	0.29	0.03 - 3.26	0.314956
	GSE13507	ILMN_1742179	Disease Specific Survival	0.87	0.03 - 27.86	0.93535
**FNDC8**	GSE5287	220499_at	Overall Survival	1.45	0.16 - 13.06	0.740284
	GSE13507	ILMN_1682221	Overall Survival	0.98	0.12 - 8.16	0.988614
	GSE13507	ILMN_1682221	Disease Specific Survival	2.26	0.11 - 45.56	0.594606
**Blood Cancer**						
**FNDC1**	GSE12417-GPL97	226930_at	Overall Survival	0.37	0.10 - 1.33	0.126236
	GSE12417-GPL570	226930_at	Overall Survival	0.88	0.27 - 2.85	0.826778
	GSE16131-GPL97	226930_at	Overall Survival	0.78	0.63 - 0.95	0.012860
	GSE2658	226930_at	Disease Specific Survival	0.85	0.69 - 1.04	0.116402
**FNDC3A**	GSE12417-GPL96	215910_s_at	Overall Survival	1.12	0.62 - 2.05	0.704536
	GSE12417-GPL96	202304_at	Overall Survival	1.25	0.86 - 1.81	0.247064
	GSE12417-GPL97	238961_s_at	Overall Survival	1.92	0.44 - 8.45	0.386565
	GSE12417-GPL97	241611_s_at	Overall Survival	2.70	0.84 - 8.63	0.093910
	GSE12417-GPL570	215910_s_at	Overall Survival	1.87	0.86 - 4.08	0.115623
	GSE12417-GPL570	202304_at	Overall Survival	1.33	0.85 - 2.07	0.213120
	GSE12417-GPL570	238961_s_at	Overall Survival	0.38	0.06 - 2.44	0.309943
	GSE12417-GPL570	241611_s_at	Overall Survival	2.29	0.49 - 10.80	0.295326
	GSE5122	202304_at	Overall Survival	0.99	0.60 - 1.63	0.978075
	GSE5122	215910_s_at	Overall Survival	0.93	0.70 - 1.24	0.616633
	GSE8970	202304_at	Overall Survival	1.42	0.80 - 2.50	0.230733
	GSE8970	215910_s_at	Overall Survival	1.22	0.85 - 1.76	0.285164
	GSE4475	202304_at	Overall Survival	1.58	0.94 - 2.67	0.084938
	GSE4475	215910_s_at	Overall Survival	3.60	1.05 - 12.30	0.041497
	E-TABM-346	215910_s_at	Overall Survival	1.03	0.60 - 1.78	0.918865

	E-TABM-346	202304_at	Event Free Survival	1.49	0.64 - 3.48	0.358875
	E-TABM-346	215910_s_at	Event Free Survival	0.99	0.61 - 1.61	0.974748
	E-TABM-346	202304_at	Overall Survival	1.94	0.81 - 4.62	0.134777
	GSE16131-GPL96	202304_at	Overall Survival	0.98	0.60 - 1.59	0.931084
	GSE16131-GPL96	215910_s_at	Overall Survival	1.08	0.91 - 1.28	0.398988
	GSE16131-GPL97	238961_s_at	Overall Survival	1.03	0.85 - 1.24	0.769930
	GSE16131-GPL97	241611_s_at	Overall Survival	1.05	0.90 - 1.24	0.532598
	GSE2658	215910_s_at	Disease Specific Survival	0.77	0.54 - 1.10	0.151066
	GSE2658	202304_at	Disease Specific Survival	0.49	0.32 - 0.76	0.001450
	GSE2658	238961_s_at	Disease Specific Survival	0.92	0.72 - 1.16	0.460040
	GSE2658	241611_s_at	Disease Specific Survival	0.97	0.79 - 1.18	0.752126
**FNDC3B**	GSE12417-GPL96	218618_s_at	Overall Survival	1.14	0.91 - 1.43	0.260621
	GSE12417-GPL97	225032_at	Overall Survival	1.13	0.87 - 1.46	0.354041
	GSE12417-GPL97	222692_s_at	Overall Survival	1.07	0.77 - 1.49	0.668754
	GSE12417-GPL97	229865_at	Overall Survival	1.17	0.91 - 1.50	0.218468
	GSE12417-GPL97	222693_at	Overall Survival	1.09	0.76 - 1.57	0.640113
	GSE12417-GPL97	242029_at	Overall Survival	1.54	0.60 - 3.96	0.36856
	GSE12417-GPL570	225032_at	Overall Survival	1.54	0.93 - 2.55	0.095537
	GSE12417-GPL570	222692_s_at	Overall Survival	1.80	1.10 - 2.94	0.018341
	GSE12417-GPL570	222693_at	Overall Survival	2.20	1.30 - 3.71	0.003167
	GSE12417-GPL570	242029_at	Overall Survival	1.76	0.86 - 3.59	0.119358
	GSE12417-GPL570	1569490_at	Overall Survival	2.62	0.71 - 9.59	0.146865
	GSE12417-GPL570	229865_at	Overall Survival	1.36	0.97 - 1.89	0.071774
	GSE12417-GPL570	218618_s_at	Overall Survival	1.37	0.98 - 1.92	0.067769
	GSE5122	218618_s_at	Overall Survival	1.35	0.85 - 2.15	0.201167
	GSE8970	218618_s_at	Overall Survival	1.03	0.76 - 1.40	0.837297
	GSE4475	218618_s_at	Overall Survival	0.93	0.59 - 1.46	0.761987
	E-TABM-346	218618_s_at	Overall Survival	1.25	0.61 - 2.54	0.540052
	E-TABM-346	218618_s_at	Event Free Survival	1.17	0.58 - 2.36	0.653477
	GSE16131-GPL96	218618_s_at	Overall Survival	1.30	0.83 - 2.04	0.2549
	GSE16131-GPL97	225032_at	Overall Survival	1.31	0.74 - 2.31	0.350281
	GSE16131-GPL97	222692_s_at	Overall Survival	1.18	0.87 - 1.61	0.274853
	GSE16131-GPL97	229865_at	Overall Survival	1.01	0.81 - 1.26	0.915584
	GSE16131-GPL97	222693_at	Overall Survival	1.00	0.78 - 1.27	0.971569
	GSE16131-GPL97	242029_at	Overall Survival	0.93	0.73 - 1.19	0.575318
	GSE2658	225032_at	Disease Specific Survival	0.52	0.29 - 0.92	0.024162
	GSE2658	222692_s_at	Disease Specific Survival	0.60	0.41 - 0.88	0.009594
	GSE2658	222693_at	Disease Specific Survival	0.78	0.52 - 1.16	0.218143
	GSE2658	218618_s_at	Disease Specific Survival	0.77	0.51 - 1.18	0.230352
	GSE2658	242029_at	Disease Specific Survival	0.97	0.81 - 1.16	0.706238
	GSE2658	1569490_at	Disease Specific Survival	1.10	0.74 - 1.64	0.624482
	GSE2658	229865_at	Disease Specific Survival	1.10	0.84 - 1.45	0.481435
**FNDC4**	GSE12417-GPL96	218843_at	Overall Survival	0.23	0.06 - 0.87	0.030692
	GSE12417-GPL570	218843_at	Overall Survival	0.87	0.21 - 3.56	0.850621

	GSE5122	218843_at	Overall Survival	0.90	0.61 - 1.34	0.610764
	GSE8970	218843_at	Overall Survival	0.89	0.41 - 1.95	0.771075
	GSE4475	218843_at	Overall Survival	2.49	0.46 - 13.55	0.290572
	E-TABM-346	218843_at	Overall Survival	0.68	0.23 - 2.05	0.492865
	E-TABM-346	218843_at	Event Free Survival	0.53	0.19 - 1.51	0.232756
	GSE16131-GPL96	218843_at	Overall Survival	1.31	0.81 - 2.11	0.270073
	GSE2658	218843_at	Disease Specific Survival	0.87	0.55 - 1.38	0.563693
**FNDC5**	GSE12417-GPL97	230646_at	Overall Survival	0.36	0.09 - 1.41	0.142104
	GSE12417-GPL97	226096_at	Overall Survival	0.63	0.12 - 3.28	0.583433
	GSE12417-GPL97	226097_at	Overall Survival	0.09	0.01 - 0.67	0.018029
	GSE12417-GPL570	226097_at	Overall Survival	0.45	0.07 - 2.78	0.386579
	GSE12417-GPL570	230646_at	Overall Survival	0.17	0.03 - 0.84	0.029424
	GSE12417-GPL570	226096_at	Overall Survival	1.28	0.34 - 4.89	0.715945
	GSE16131-GPL97	226096_at	Overall Survival	1.15	0.78 - 1.69	0.479834
	GSE16131-GPL97	226097_at	Overall Survival	0.81	0.50 - 1.32	0.398332
	GSE16131-GPL97	230646_at	Overall Survival	0.69	0.39 - 1.20	0.189115
	GSE2658	226097_at	Disease Specific Survival	0.51	0.31 - 0.83	0.00749
	GSE2658	230646_at	Disease Specific Survival	0.64	0.42 - 0.98	0.037867
	GSE2658	226096_at	Disease Specific Survival	0.79	0.55 - 1.12	0.188239
**FNDC6**	GSE12417-GPL97	228575_at	Overall Survival	1.41	0.27 - 7.42	0.687376
	GSE12417-GPL570	228575_at	Overall Survival	0.28	0.05 - 1.47	0.13193
	GSE16131-GPL97	228575_at	Overall Survival	1.05	0.82 - 1.35	0.680191
	GSE2658	228575_at	Disease Specific Survival	0.72	0.55 - 0.92	0.010312
**FNDC7**	GSE12417-GPL97	240837_at	Overall Survival	1.03	0.17 - 6.34	0.976891
	GSE12417-GPL570	240837_at	Overall Survival	1.71	0.24 - 12.14	0.59193
	GSE16131-GPL97	240837_at	Overall Survival	1.21	0.96 - 1.52	0.109998
	GSE2658	240837_at	Disease Specific Survival	0.92	0.69 - 1.23	0.574838
**FNDC8**	GSE12417-GPL96	220499_at	Overall Survival	0.47	0.12 - 1.87	0.286634
	GSE12417-GPL570	220499_at	Overall Survival	0.32	0.05 - 2.12	0.239044
	GSE5122	220499_at	Overall Survival	1.27	0.93 - 1.75	0.132785
	GSE8970	220499_at	Overall Survival	0.74	0.44 - 1.23	0.249101
	GSE4475	220499_at	Overall Survival	0.97	0.20 - 4.74	0.965113
	E-TABM-346	220499_at	Event Free Survival	1.28	0.84 - 1.95	0.256798
	E-TABM-346	220499_at	Overall Survival	1.33	0.83 - 2.12	0.233377
	GSE16131-GPL96	220499_at	Overall Survival	1.07	0.80 - 1.44	0.627334
	GSE2658	220499_at	Disease Specific Survival	0.80	0.60 - 1.07	0.139664
**Brain Cancer**						
**FNDC1**	GSE4271-GPL97	226930_at	Overall Survival	1.00	0.81 - 1.22	0.968863
	GSE7696	226930_at	Overall Survival	0.76	0.50 - 1.18	0.221402
	GSE4412-GPL97	226930_at	Overall Survival	1.31	1.00 - 1.71	0.048686
	GSE16581	226930_at	Overall Survival	0.54	0.20 - 1.47	0.229395
**FNDC3A**	GSE4271-GPL96	202304_at	Overall Survival	0.91	0.48 - 1.72	0.770460
	GSE4271-GPL96	215910_s_at	Overall Survival	0.86	0.61 - 1.23	0.413508
	GSE4271-GPL97	238961_s_at	Overall Survival	1.17	0.82 - 1.68	0.392104

	GSE4271-GPL97	241611_s_at	Overall Survival	0.88	0.69 - 1.13	0.329405
	GSE7696	202304_at	Overall Survival	1.00	0.74 - 1.37	0.977152
	GSE7696	238961_s_at	Overall Survival	0.37	0.04 - 3.16	0.363487
	GSE7696	241611_s_at	Overall Survival	1.62	0.45 - 5.86	0.458942
	GSE7696	215910_s_at	Overall Survival	0.57	0.17 - 1.88	0.355036
	MGH-glioma	38649_at	Overall Survival	0.79	0.41 - 1.50	0.467319
	GSE4412-GPL96	202304_at	Overall Survival	0.71	0.31 - 1.63	0.416691
	GSE4412-GPL96	215910_s_at	Overall Survival	0.65	0.46 - 0.92	0.015105
	GSE4412-GPL97	238961_s_at	Overall Survival	1.05	0.70 - 1.56	0.819500
	GSE4412-GPL97	241611_s_at	Overall Survival	1.00	0.75 - 1.33	0.997149
	GSE16581	215910_s_at	Overall Survival	1.21	0.19 - 7.53	0.838736
	GSE16581	238961_s_at	Overall Survival	0.09	0.00 - 6.55	0.270332
	GSE16581	241611_s_at	Overall Survival	0.32	0.04 - 2.36	0.266392
	GSE16581	202304_at	Overall Survival	1.04	0.03 - 31.97	0.980655
**FNDC3B**	GSE4271-GPL96	218618_s_at	Overall Survival	1.43	0.97 - 2.10	0.067786
	GSE4271-GPL97	222692_s_at	Overall Survival	1.49	1.11 - 2.01	0.008227
	GSE4271-GPL97	229865_at	Overall Survival	1.36	0.96 - 1.92	0.079678
	GSE4271-GPL97	222693_at	Overall Survival	1.32	0.95 - 1.83	0.100977
	GSE4271-GPL97	242029_at	Overall Survival	1.25	0.94 - 1.67	0.124403
	GSE4271-GPL97	225032_at	Overall Survival	1.45	0.93 - 2.26	0.104038
	GSE7696	225032_at	Overall Survival	0.93	0.63 - 1.38	0.710968
	GSE7696	222692_s_at	Overall Survival	0.88	0.63 - 1.23	0.450123
	GSE7696	222693_at	Overall Survival	0.79	0.54 - 1.15	0.223305
	GSE7696	242029_at	Overall Survival	0.92	0.55 - 1.54	0.738171
	GSE7696	1569490_at	Overall Survival	1.00	0.29 - 3.41	0.997059
	GSE7696	229865_at	Overall Survival	0.70	0.38 - 1.30	0.25903
	GSE7696	218618_s_at	Overall Survival	0.99	0.72 - 1.37	0.96081
	GSE4412-GPL96	218618_s_at	Overall Survival	1.40	0.92 - 2.12	0.112305
	GSE4412-GPL97	229865_at	Overall Survival	1.50	0.95 - 2.38	0.082628
	GSE4412-GPL97	222693_at	Overall Survival	1.03	0.76 - 1.39	0.871473
	GSE4412-GPL97	242029_at	Overall Survival	1.13	0.85 - 1.51	0.388749
	GSE4412-GPL97	225032_at	Overall Survival	1.47	0.87 - 2.47	0.152295
	GSE4412-GPL97	222692_s_at	Overall Survival	1.17	0.87 - 1.57	0.303637
	GSE16581	242029_at	Overall Survival	0.58	0.14 - 2.38	0.449015
	GSE16581	225032_at	Overall Survival	1.25	0.08 - 20.68	0.877835
	GSE16581	1569490_at	Overall Survival	1.61	0.03 - 78.54	0.809817
	GSE16581	229865_at	Overall Survival	2.26	0.27 - 18.63	0.449467
	GSE16581	222693_at	Overall Survival	0.71	0.08 - 6.44	0.763694
	GSE16581	218618_s_at	Overall Survival	1.39	0.12 - 15.66	0.79221
	GSE16581	222692_s_at	Overall Survival	0.47	0.06 - 4.07	0.495747
**FNDC4**	GSE4271-GPL96	218843_at	Overall Survival	0.82	0.58 - 1.17	0.272063
	GSE7696	218843_at	Overall Survival	0.83	0.36 - 1.93	0.669769
	GSE4412-GPL96	218843_at	Overall Survival	1.57	1.00 - 2.45	0.048195
	GSE16581	218843_at	Overall Survival	0.99	0.05 - 18.87	0.994805

**FNDC5**	GSE4271-GPL97	226096_at	Overall Survival	0.83	0.67 - 1.04	0.107581
	GSE4271-GPL97	226097_at	Overall Survival	1.03	0.68 - 1.56	0.883576
	GSE4271-GPL97	230646_at	Overall Survival	1.43	0.58 - 3.51	0.440563
	GSE7696	226097_at	Overall Survival	1.06	0.21 - 5.39	0.941442
	GSE7696	230646_at	Overall Survival	1.28	0.26 - 6.35	0.759161
	GSE7696	226096_at	Overall Survival	0.89	0.53 - 1.49	0.646035
	GSE4412-GPL97	226097_at	Overall Survival	0.58	0.20 - 1.64	0.301398
	GSE4412-GPL97	230646_at	Overall Survival	3.32	1.11 - 9.92	0.031731
	GSE4412-GPL97	226096_at	Overall Survival	1.10	0.87 - 1.38	0.43734
	GSE16581	226096_at	Overall Survival	1.61	0.19 - 13.43	0.66183
	GSE16581	226097_at	Overall Survival	0.55	0.02 - 15.58	0.72392
	GSE16581	230646_at	Overall Survival	54.9	0.52 - 5846	0.092604
**FNDC6**	GSE4271-GPL97	228575_at	Overall Survival	0.95	0.63 - 1.43	0.819729
	GSE7696	228575_at	Overall Survival	1.01	0.21 - 4.85	0.991576
	GSE4412-GPL97	228575_at	Overall Survival	1.47	0.90 - 2.41	0.125053
	GSE16581	228575_at	Overall Survival	3.31	0.25 - 42.99	0.360356
**FNDC7**	GSE4271-GPL97	240837_at	Overall Survival	1.43	0.98 - 2.08	0.062706
	GSE7696	240837_at	Overall Survival	1.21	0.57 - 2.58	0.622259
	GSE4412-GPL97	240837_at	Overall Survival	1.47	1.01 - 2.12	0.042891
	GSE16581	240837_at	Overall Survival	18.0	0.49 - 671	0.116281
**FNDC8**	GSE4271-GPL96	220499_at	Overall Survival	1.00	0.66 - 1.51	0.999175
	GSE7696	220499_at	Overall Survival	2.10	0.53 - 8.32	0.290771
	GSE4412-GPL96	220499_at	Overall Survival	1.18	0.77 - 1.80	0.448594
	GSE16581	220499_at	Overall Survival	18.8	0.54 - 657	0.105846
**Skin Cancer**						
**FNDC1**	GSE19234	226930_at	Overall Survival	1.06	0.76 - 1.47	0.732520
**FNDC3A**	GSE19234	215910_s_at	Overall Survival	4.06	1.40 - 11.80	0.010107
	GSE19234	202304_at	Overall Survival	1.76	0.69 - 4.53	0.240145
	GSE19234	238961_s_at	Overall Survival	1.41	0.53 - 3.73	0.490007
	GSE19234	241611_s_at	Overall Survival	0.91	0.43 - 1.92	0.800243
**FNDC3B**	GSE19234	242029_at	Overall Survival	0.69	0.21 - 2.26	0.537686
	GSE19234	225032_at	Overall Survival	1.56	0.56 - 4.37	0.397884
	GSE19234	1569490_at	Overall Survival	0.75	0.38 - 1.48	0.40203
	GSE19234	229865_at	Overall Survival	3.22	1.25 - 8.28	0.015536
	GSE19234	218618_s_at	Overall Survival	1.16	0.53 - 2.58	0.707213
	GSE19234	222692_s_at	Overall Survival	1.94	0.89 - 4.26	0.096869
	GSE19234	222693_at	Overall Survival	1.95	0.92 - 4.11	0.081098
**FNDC4**	GSE19234	218843_at	Overall Survival	2.04	1.10 - 3.78	0.02409
**FNDC5**	GSE19234	226097_at	Overall Survival	0.85	0.43 - 1.69	0.650662
	GSE19234	230646_at	Overall Survival	0.84	0.55 - 1.29	0.427695
	GSE19234	226096_at	Overall Survival	1.59	0.32 - 7.79	0.568206
**FNDC6**	GSE19234	228575_at	Overall Survival	0.93	0.61 - 1.41	0.72045
**FNDC7**	GSE19234	240837_at	Overall Survival	1.83	0.72 - 4.60	0.201848
**FNDC8**	GSE19234	220499_at	Overall Survival	0.73	0.27 - 2.00	0.546013

HR, hazard ratio; CI, confidence interval. All of the data were obtained from the PrognoScan database. The data with statistical significance were marked in red.
