# Supplementary material for: Expression and prognosis analyses of the fibronectin type-III domain-containing (FNDC) protein family in human cancers: A Review
Source: Medicine (Baltimore). 2022 Dec 9;101(49):e31854. doi: 10.1097/MD.0000000000031854 (PMC9750624; doi:10.1097/MD.0000000000031854)
Supplement: Supplementary file 11 [file medi-101-e31854-s011.pdf]

Table H. Survival analyses of FNDC family in different St Gallen subtypes of breast cancer.

Gene	Affymetrix ID	Survival outcome	Basal-like		Lumina A		Lumina B		HER2+	
			HR (95% CI)	p-value	HR (95% CI)	p-value	HR (95% CI)	p-value	HR (95% CI)	p-value
**FNDC1**	226930_at	RFS	1.02 (0.75 - 1.38)	0.9191	0.81 (0.64 - 1.04)	0.0969	0.98 (0.73 - 1.32)	0.8895	1.12 (0.72 - 1.73)	0.6158
		OS	0.99 (0.62 - 1.57)	0.9542	0.93 (0.58 - 1.5)	0.7678	1.27 (0.68 - 2.36)	0.4475	1.37 (0.69 - 2.74)	0.3676
		DMFS	1.49 (0.93 - 2.4)	0.0946	1.13 (0.69 - 1.87)	0.6272	1.06 (0.6 - 1.89)	0.8401	2.07 (1.1 - 3.88)	0.0214
		PPS	0.51 (0.22 - 1.16)	0.1011	0.89 (0.5 - 1.58)	0.6919	1.74 (0.82 - 3.66)	0.1421	0.79 (0.34 - 1.83)	0.5825
**FNDC3A**	218618_s_at	RFS	0.9 (0.72 - 1.12)	0.3541	0.79 (0.67 - 0.93)	0.0048	0.93 (0.78 - 1.11)	0.4021	0.86 (0.6 - 1.23)	0.409
		OS	0.68 (0.46 - 1)	0.0474	0.74 (0.53 - 1.02)	0.064	0.83 (0.58 - 1.18)	0.2916	0.65 (0.36 - 1.16)	0.1411
		DMFS	0.71 (0.52 - 0.97)	0.0305	0.74 (0.57 - 0.97)	0.0276	0.83 (0.63 - 1.1)	0.1995	0.87 (0.53 - 1.42)	0.5744
		PPS	0.89 (0.52 - 1.55)	0.691	1.33 (0.93 - 1.9)	0.1137	1.49 (0.97 - 2.28)	0.0645	1 (0.47 - 2.11)	0.999
	215910_s_at	RFS	0.85 (0.68 - 1.07)	0.1629	0.71 (0.61 - 0.84)	5.8e-5	0.81 (0.68 - 0.96)	0.0174	0.61 (0.42 - 0.87)	0.0063
		OS	0.9 (0.61 - 1.31)	0.5708	0.79 (0.57 - 1.08)	0.133	0.69 (0.49 - 0.98)	0.0378	0.58 (0.32 - 1.03)	0.0588
		DMFS	0.93 (0.68 - 1.27)	0.6594	0.83 (0.64 - 1.08)	0.1636	0.93 (0.71 - 1.23)	0.6301	0.66 (0.4 - 1.09)	0.1012
		PPS	1.47 (0.84 - 2.58)	0.1736	1.04 (0.73 - 1.49)	0.821	0.83 (0.54 - 1.27)	0.3882	1.27 (0.6 - 2.68)	0.5268
	241611_s_at	RFS	0.82 (0.61 - 1.12)	0.2161	1 (0.78 - 1.27)	0.9783	1.05 (0.78 - 1.42)	0.7501	0.75 (0.48 - 1.17)	0.2047
		OS	0.53 (0.33 - 0.86)	0.0089	0.79 (0.49 - 1.28)	0.3313	1.11 (0.6 - 2.06)	0.7353	1.03 (0.52 - 2.03)	0.9403
		DMFS	0.74 (0.46 - 1.18)	0.2021	0.83 (0.49 - 1.38)	0.4605	1.28 (0.72 - 2.3)	0.3965	0.9 (0.49 - 1.63)	0.7204
		PPS	0.47 (0.21 - 1.08)	0.0697	0.99 (0.56 - 1.76)	0.9727	1.11 (0.53 - 2.3)	0.7867	1.25 (0.59 - 2.69)	0.9556
**FNDC3B**	1559975_at	RFS	1.15 (0.92 - 1.44)	0.2113	1.16 (0.99 - 1.37)	0.0721	1.34 (1.12 - 1.6)	0.0011	1.08 (0.76 - 1.54)	0.6614
		OS	0.8 (0.55 - 1.18)	0.2593	0.87 (0.63 - 1.19)	0.3755	1.21 (0.86 - 1.72)	0.2735	0.96 (0.55 - 1.7)	0.9014
		DMFS	0.69 (0.51 - 0.95)	0.0221	1.16 (0.89 - 1.5)	0.269	1.05 (0.79 - 1.39)	0.7489	1.47 (0.89 - 2.42)	0.1289
		PPS	0.86 (0.49 - 1.51)	0.5992	1.26 (0.88 - 1.81)	0.2046	1.45 (0.95 - 2.23)	0.0839	1.18 (0.56 - 2.48)	0.6649
	222692_s_at	RFS	0.81 (0.59 - 1.1)	0.167	1.11 (0.87 - 1.42)	0.3847	1.15 (0.85 - 1.55)	0.374	1.01 (0.65 - 1.56)	0.9596
		OS	0.88 (0.56 - 1.4)	0.5959	2.07 (1.25 - 3.43)	0.0037	0.85 (0.46 - 1.58)	0.6124	0.74 (0.37 - 1.47)	0.3819
		DMFS	0.69 (0.43 - 1.11)	0.1271	1.03 (0.62 - 1.7)	0.9096	0.81 (0.46 - 1.46)	0.4874	1.02 (0.56 - 1.85)	0.9523
		PPS	0.88 (0.4 – 1.94)	0.7523	2.31 (1.29 - 4.11)	0.0036	1.44 (0.69 - 3)	0.3307	0.91 (0.39 - 2.11)	0.8322
	222693_at	RFS	0.73 (0.54 - 1)	0.0478	0.67 (0.53 - 0.86)	0.0013	0.69 (0.51 - 0.94)	0.0175	0.59 (0.38 - 0.91)	0.0168
		OS	0.49 (0.3 - 0.79)	0.0032	1.01 (0.63 - 1.62)	0.9722	0.8 (0.43 - 1.47)	0.4685	0.95 (0.48 - 1.87)	0.8737
		DMFS	0.71 (0.45 - 1.14)	0.1583	1.67 (0.99 - 2.8)	0.05	1.03 (0.58 - 1.84)	0.9252	0.8 (0.44 - 1.46)	0.4618
		PPS	0.87 (0.39 - 1.95)	0.7377	1.2 (0.68 - 2.14)	0.5251	1.15 (0.55 - 2.38)	0.7101	1.59 (0.68 - 3.69)	0.2788
	225032_at	RFS	1.07 (0.79 - 1.46)	0.6531	0.79 (0.62 - 1.01)	0.0576	0.89 (0.66 - 1.21)	0.4625	0.94 (0.61 - 1.45)	0.7783
		OS	0.85 (0.54 - 1.35)	0.4943	1.43 (0.89 - 2.31)	0.1385	0.83 (0.45 - 1.53)	0.55	1.08 (0.54 - 2.14)	0.8295
		DMFS	1.44 (0.9 - 2.3)	0.1302	1.72 (1.03 - 2.88)	0.0357	0.85 (0.48 - 1.53)	0.5963	1.19 (0.65 - 2.17)	0.5736
		PPS	0.88 (0.4 - 1.93)	0.747	1.08 (0.61 - 1.91)	0.7833	1.33 (0.64 - 2.77)	0.4398	0.75 (0.32 - 1.74)	0.5001
**FNDC4**	218843_at	RFS	1.2 (0.96 - 1.5)	0.1054	0.75 (0.64 - 0.89)	0.0006	0.93 (0.78 - 1.11)	0.4069	1.06 (0.75 - 1.52)	0.7309
		OS	1.6 (1.09 - 2.36)	0.0157	0.74 (0.54 - 1.02)	0.063	0.93 (0.65 - 1.31)	0.6705	1.16 (0.66 - 2.05)	0.6069
		DMFS	1.15 (0.84 - 1.57)	0.3848	0.79 (0.61 - 1.03)	0.0828	1.25 (0.94 - 1.65)	0.1224	1.13 (0.69 - 1.85)	0.6335
		PPS	1.36 (0.78 - 2.36)	0.2768	0.86 (0.6 - 1.23)	0.4116	0.9 (0.59 - 1.39)	0.637	0.67 (0.31 - 1.42)	0.2937
**FNDC5**	226096_at	RFS	0.88 (0.65 - 1.19)	0.4038	0.86 (0.68 - 1.1)	0.2366	0.92 (0.68 - 1.24)	0.5682	1.08 (0.69 - 1.66)	0.7437
		OS	0.85 (0.54 - 1.36)	0.5038	0.78 (0.48 - 1.25)	0.2991	0.56 (0.3 - 1.06)	0.0719	1.3 (0.65 - 2.57)	0.4545
		DMFS	0.53 (0.32 - 0.86)	0.0085	0.92 (0.56 - 1.53)	0.7614	1.13 (0.63 - 2.02)	0.6796	1.41 (0.77 - 2.57)	0.2663
		PPS	0.85 (0.38 - 1.87)	0.6765	0.96 (0.55 - 1.67)	0.8856	0.56 (0.27 - 1.18)	0.1234	2.06 (0.87 - 4.87)	0.0933
	226097_at	RFS	0.54 (0.39 - 0.74)	9.8e-5	0.82 (0.64 - 1.04)	0.1051	0.74 (0.54 - 1)	0.0471	0.98 (0.64 - 1.52)	0.9456

		OS	1.2 (0.75 - 1.9)	0.4467	1.17 (0.73 - 1.89)	0.5203	1.02 (0.55 - 1.89)	0.9488	1.15 (0.58 - 2.29)	0.6852
		DMFS	0.7 (0.44 - 1.12)	0.1318	0.91 (0.55 - 1.51)	0.7247	1.01 (0.57 - 1.8)	0.9712	1.25 (0.68 - 2.29)	0.4689
		PPS	1.41 (0.64 - 3.11)	0.3976	1.1 (0.63 - 1.92)	0.7366	1.29 (0.62 - 2.69)	0.4975	1.34 (0.57 - 3.13)	0.4971
**FNDC6**	228575_at	RFS	0.68 (0.5 - 0.93)	0.0139	0.91 (0.72 - 1.16)	0.4468	0.68 (0.5 - 0.92)	0.0123	1.1 (0.71 - 1.71)	0.6628
		OS	1.04 (0.66 - 1.66)	0.8565	1.02 (0.64 - 1.64)	0.9248	1.11 (0.6 - 2.06)	0.7328	2.23 (1.08 - 4.61)	0.026
		DMFS	0.99 (0.62 - 1.57)	0.9607	1.58 (0.94 - 2.64)	0.0796	1.34 (0.75 - 2.41)	0.3189	1.64 (0.89 - 3.03)	0.1107
		PPS	3.01 (1.28 - 7.06)	0.0087	1.18 (0.67 - 2.08)	0.5644	1.03 (0.49 - 2.14)	0.9445	1.69 (0.73 - 3.92)	0.2192
**FNDC7**	240837_at	RFS	0.81 (0.59 - 1.1)	0.1691	0.94 (0.74 - 1.19)	0.5949	0.92 (0.68 - 1.25)	0.6099	1.22 (0.79 - 1.89)	0.368
		OS	1.18 (0.74 - 1.87)	0.4859	1.12 (0.7 - 1.8)	0.6269	1.45 (0.78 - 2.7)	0.2345	1.19 (0.6 - 2.36)	0.6107
		DMFS	1.1 (0.69 - 1.74)	0.7003	1.05 (0.64 -1.74)	0.8412	1.51 (0.84 - 2.71)	0.164	1.09 (0.6 - 1.99)	0.7673
		PPS	0.78 (0.35 - 1.71)	0.5291	0.85 (0.49 - 1.49)	0.5693	1.59 (0.75 - 3.38)	0.2247	1.37 (0.59 - 3.19)	0.4672
**FNDC8**	220499_at	RFS	1.01 (0.81 - 1.26)	0.9152	0.92 (0.78 - 1.09)	0.3382	0.93 (0.78 - 1.11)	0.4302	0.88 (0.62 - 1.26)	0.4945
		OS	1.23 (0.84 - 1.8)	0.276	0.86 (0.62 - 1.18)	0.3452	1.09 (0.77 - 1.54)	0.6438	1.18 (0.67 - 2.08)	0.5702
		DMFS	1.34 (0.98 - 1.84)	0.0619	0.96 (0.74 - 1.25)	0.7698	1.26 (0.95 - 1.67)	0.1023	0.97 (0.59 - 1.6)	0.9151
		PPS	1.24 (0.71 - 2.16)	0.4442	0.84 (0.58 - 1.2)	0.3359	1.08 (0.71 - 1.65)	0.7132	1.03 (0.49 - 2.18)	0.9308

HR, hazard ratio; CI, confidence interval; OS, overall survival; RFS, relapse free survival; DMFS, distant metastasis free survival; PPS, post progression survival. All of the data were obtained from the Kaplan-Meier Plotter database. The data with statistical significance were marked in red.
